# Nicotinamide and pyridoxine stimulate muscle stem cell expansion and enhance regenerative capacity during aging

**DOI:** 10.1172/JCI163648

**Published:** 2024-11-12

**Authors:** Sara Ancel, Joris Michaud, Eugenia Migliavacca, Charline Jomard, Aurélie Fessard, Pauline Garcia, Sonia Karaz, Sruthi Raja, Guillaume E. Jacot, Thibaut Desgeorges, José L. Sánchez-García, Loic Tauzin, Yann Ratinaud, Benjamin Brinon, Sylviane Métairon, Lucas Pinero, Denis Barron, Stephanie Blum, Leonidas G. Karagounis, Ramin Heshmat, Afshin Ostovar, Farshad Farzadfar, Isabella Scionti, Rémi Mounier, Julien Gondin, Pascal Stuelsatz, Jerome N. Feige

**Affiliations:** 1 Nestlé Institute of Health Sciences, Nestlé Research, Lausanne, Switzerland.; 2 School of Life Sciences, Ecole Polytechnique Fédérale de Lausanne, Lausanne, Switzerland.; 3 Institut NeuroMyoGène, Physiopathologie et Génétique du Neurone et du Muscle, Université Claude Bernard Lyon 1, CNRS UMR5261, INSERM U1315, Lyon, France.; 4 Nestlé Institute of Food Safety and Analytical Sciences, Nestlé Research, Lausanne, Switzerland.; 5 Translational Research, Nestlé Health Science, Lausanne, Switzerland.; 6 Mary MacKillop Institute for Health Research, Australian Catholic University, Melbourne, Australia.; 7 Chronic Diseases Research Center, Endocrinology and Metabolism Population Sciences Institute, Tehran University of Medical Sciences, Tehran, Iran.

**Keywords:** Muscle biology, Stem cells, Adult stem cells, Epidemiology, Skeletal muscle

## Abstract

Skeletal muscle relies on resident muscle stem cells (MuSCs) for growth and repair. Aging and muscle diseases impair MuSC function, leading to stem cell exhaustion and regenerative decline that contribute to the progressive loss of skeletal muscle mass and strength. In the absence of clinically available nutritional solutions specifically targeting MuSCs, we used a human myogenic progenitor high-content imaging screen of natural molecules from food to identify nicotinamide (NAM) and pyridoxine (PN) as bioactive nutrients that stimulate MuSCs and have a history of safe human use. NAM and PN synergize via CK1-mediated cytoplasmic β-catenin activation and AKT signaling to promote amplification and differentiation of MuSCs. Oral treatment with a combination of NAM and PN accelerated muscle regeneration in vivo by stimulating MuSCs, increased muscle strength during recovery, and overcame MuSC dysfunction and regenerative failure during aging. Levels of NAM and bioactive PN spontaneously declined during aging in model organisms and interindependently associated with muscle mass and walking speed in a cohort of 186 aged people. Collectively, our results establish the NAM/PN combination as a nutritional intervention that stimulates MuSCs, enhances muscle regeneration, and alleviates age-related muscle decline with a direct opportunity for clinical translation.

## Abstract 2

Ancel et al identify NAM/PN as bioactive nutrients which synergistically stimulate muscle stem cells and accelerate regeneration during muscle recovery and aging.

## Graphical abstract

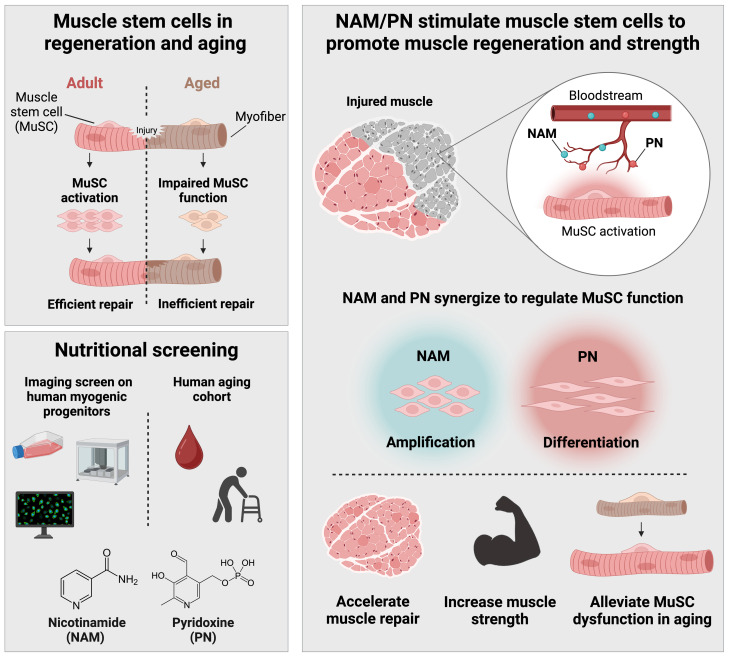

## Introduction

Skeletal muscle is a remarkably plastic tissue that adapts structurally and functionally to lifestyle or external stimuli, such as exercise, disuse, or injury. Resident PAX7-expressing muscle stem cells (MuSCs), also known as satellite cells, drive tissue repair during regeneration and contribute directly to muscle growth and long-term muscle maintenance (1, 2). Under homeostatic conditions, MuSCs are maintained in a quiescent state by signals from the local niche that keep them cell-cycle arrested (3). MuSCs are essential to repair myofibers after damage, for which mechanical and inflammatory niche-derived signals stimulate their proliferation and differentiation while also ensuring self-renewal to replenish the pool (1). MuSCs also contribute to myonuclei turnover during homeostasis (4) and can fuse to myofibers to maintain myonuclear domains and support resistance training induced muscle hypertrophy (5, 6). The regulation of MuSC metabolism is critical for their fate as activation and proliferation increase metabolic requirements (7, 8). While changes in macronutrient availability have been shown to influence the ability of MuSCs to support regeneration and exercise adaptation (9, 10), the understanding of which individual nutrients from food regulate MuSCs is limited.

During aging, skeletal muscle gradually loses its ability to regenerate and muscle mass and strength decline, a condition clinically known as sarcopenia. Sarcopenia is a complex and multifactorial process, involving multiple cellular mechanisms, such as impaired neuromuscular junction transmission, myofiber mitochondrial dysfunction, and oxidative stress/inflammation (11), and lifestyle factors linked to nutrition and physical activity (12). The number and regenerative capacity of MuSCs declines during aging and sarcopenia both in animal models and in humans (13, 14). This decline involves a complex interplay of cell-autonomous and niche-dependent mechanisms where MuSC epigenetic and metabolic perturbations cross-talk with altered structural and extracellular signals from myofibers, accessory cells, and systemic blood supply (14–21).

Several proof-of-concept preclinical studies have shown that age-related MuSC dysfunction can be reversed with blockade of prostaglandin degradation or p38 and JAK/STAT signaling, NAD^+^ precursors, or activators of autophagy, which restore regenerative capacity and improve muscle strength or performance (22–27). While genetic ablation of MuSCs has shown their absolute requirement for muscle regeneration and myofiber recovery (4, 28), life-long MuSC depletion in sedentary mice did not aggravate the age-related loss of muscle mass (29), suggesting that myonuclei accretion via MuSCs may not be a primary driver of sarcopenia. However, functional capacity does not decline linearly during aging in humans, where recovery from acute events is important and mobilizes MuSCs. In particular, microinjuries caused by physical activity and traumatic events such as surgeries or falls cause a transient decline in muscle mass and function, with slow and often submaximal recovery during aging (30, 31). This critical window represents an opportunity to accelerate MuSC-mediated muscle repair and prevent long-term aggravation of sarcopenia.

While pharmacological strategies to modulate MuSCs are emerging preclinically, their clinical use is often limited by the lack of safety and toxicology studies supporting clinical use and the requirement of regulatory approvals as drugs. Here, we used a high-content imaging screen of a library of natural bioactive molecules and food-derived nutrients on human myogenic progenitors (hMPs) to identify nutritional molecules targeting MuSCs. Using this approach, we discovered that nicotinamide (NAM) and pyridoxine (PN) are potent nutrients that signal through CK1/β-catenin and AKT to stimulate MuSC proliferation and induce their differentiation. Oral in vivo treatment with the NAM/PN combination enhanced MuSC expansion, increased strength recovery, and accelerated myofiber repair in young and aged mice as well as in primary myogenic cells from aged and geriatric donors. Given that circulating levels of endogenous NAM and bioactive PN are low in older people with reduced muscle mass and function and that both NAM and PN have a history of safe human use, our results provide a therapeutic solution ready for human clinical use to stimulate muscle regeneration and mitigate age-associated muscle decline.

## Results

### A high-content imaging screen identifies NAM and PN as myogenic activators in primary hMPs.

**Figure 1:**
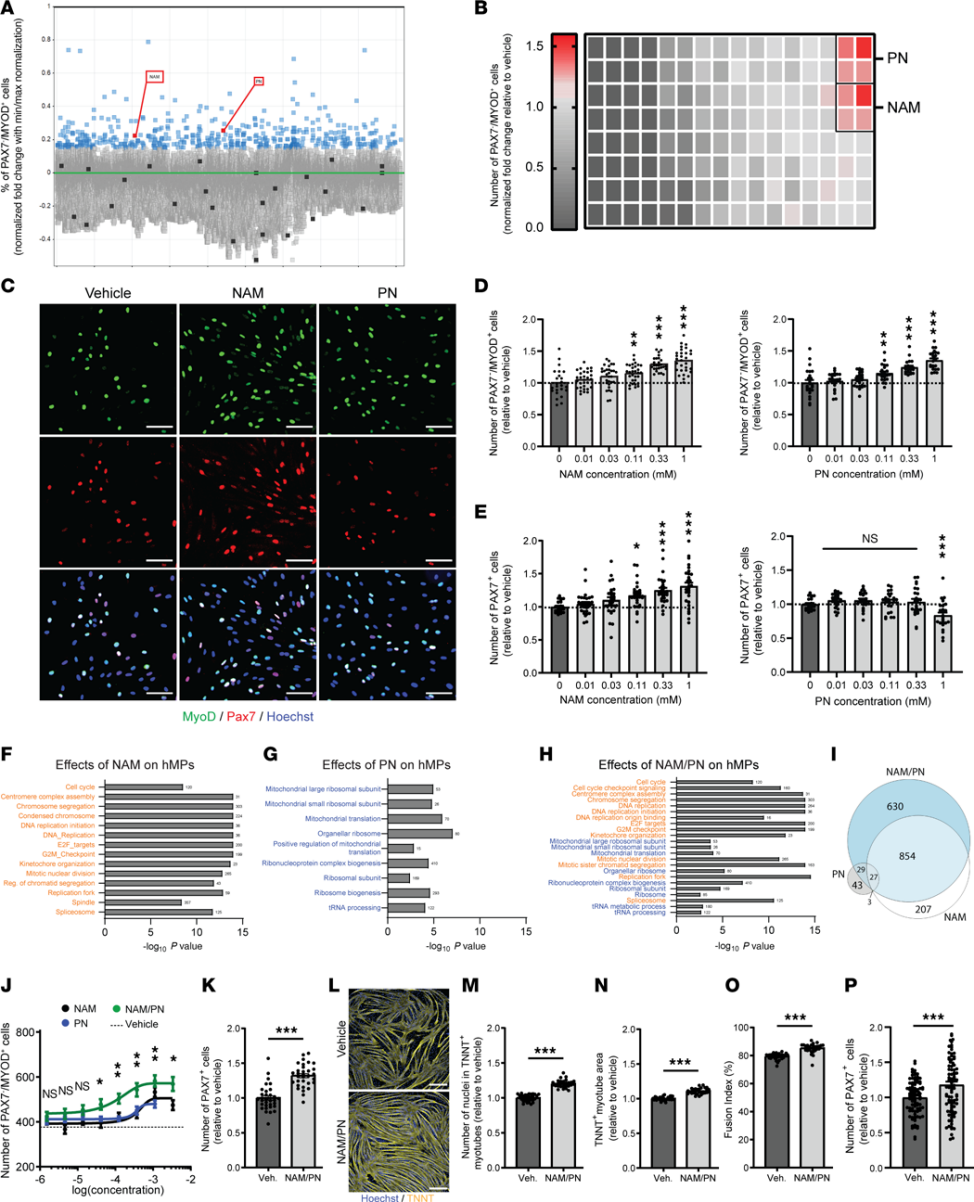
A high-content screen identifies NAM and PN as activators of hMP amplification and differentiation. (**A**) Percentage of PAX7^–^MYOD^+^ hMPs after 72-hour treatment with 50,000 bioactive molecules. Min-max normalization between negative (black line) and positive control (LY363947, green line) was performed. Blue, compounds with normalized effect size >15%; black, GRAS molecules; red, GRAS hits. (**B**) Relative number of PAX7^–^MYOD^+^ cells in hMPs treated with GRAS-classified molecules. *n* = 2–4 cell culture replicates from *N* = 2 donors. (**C**) Representative images of hMPs treated with vehicle, NAM, or PN for 72 hours. Scale bar: 100 μm. (**D** and **E**) Dose response of NAM and PN on the relative number of PAX7^–^MYOD^+^ (**D**) and PAX7^+^ (**E**) hMPs treated with vehicle, NAM, or PN for 72 hours. *n* ≥ 21 cell culture replicates from *N* = 2 donors. (**F**–**H**) Gene set enrichment analysis of upregulated gene sets in hMPs treated with NAM (**F**), PN (**G**), or the NAM/PN combination (**H**) compared with vehicle. False discovery rate, 10%. *N* = 5 donors. (**I**) Venn diagram of upregulated genes using 5% FDR multiple testing correction. (**J**) Dose response of NAM, PN, and the NAM/PN combination on PAX7^–^MYOD^+^ hMPs. *n* ≥ 6 cell culture replicates from 1 donor. (**K**) Quantification of PAX7^+^ hMPs after vehicle and NAM/PN combination treatment. *n* ≥ 32 cell culture replicates from 1 donor. (**L**–**O**) Representative images (**L**) and quantification of nuclei within myotubes (**M**), myotube area (**N**), and fusion index (**O**) in hMPs treated with vehicle or NAM/PN combination during proliferation and differentiation. Scale bar: 500 μm. *n* ≥ 28 cell culture replicates for each condition from 1 donor. (**P**) Quantification of PAX7^+^ hMPs after vehicle and NAM/PN combination treatment after myotube differentiation induction. *n* ≥ 92 cell culture replicates from 1 donor. Data are shown as the mean ± SEM. *P < 0.05; **P < 0.01; ***P < 0.001 with 1-way ANOVA followed by post hoc Dunnett’s (**D** and **E**) or Tukey’s (**J**) multiple comparison test and 2-tailed unpaired Student’s *t* tests (**K** and **M**–**P**).

To discover new natural bioactive nutrients that modulate myogenic progenitor function with a relevance for clinical translation, we developed a robust automated high-content imaging screen using primary hMP cells isolated from quadriceps muscles and validated for purity by >99% expression of the myogenic marker CD56 (Supplemental Figure 1A; supplemental material available online with this article; https://doi.org/10.1172/JCI163648DS1). We used hMPs from 2 male and 4 female representative donors who were selected based on purity and myogenic function (donors A, B, C, D, I, and J; Supplemental Table 1). Over 50,000 natural bioactive molecules and plant extracts were screened on a representative hMP donor for amplification of MYOD^+^ cells (Figure 1A and Supplemental Figure 1B). In this primary screen, 534 compounds passed the cutoff criteria of increasing by at least 15% the number of differentiating PAX7^–^MYOD^+^ cells (Figure 1A, blue data points, and Supplemental Table 2). Because our focus was on molecules with rapid translational potential, we refined our selection to FDA-approved molecules and identified NAM and PN (Figure 1A, red data points) as the most potent and generally recognized as safe (GRAS) inducers of MYOD in hMPs, with both molecules increasing by approximately 35% the number of PAX7^–^MYOD^+^ cells (Figure 1B).

To confirm and deconvolute these results, we analyzed the dose-response relationship of NAM and PN on MYOD and extended the analysis to PAX7. NAM and PN increased the number of MYOD^+^ hMPs starting at 110 μM (Figure 1, C and D), and this effect was conserved across the 3 other young hMP donors (Supplemental Figure 1C). Only NAM concomitantly increased the number of PAX7^+^ and Ki67^+^ cells (Figure 1E and Supplemental Figure 1, D–F). The effects of NAM on the number of MYOD^+^ cells resulted primarily from early effects on proliferation, as the relative number of MYOD^+^ cells normalized to total or proliferating cell number was only modestly affected by NAM (Supplemental Figure 1, G and H). In contrast, PN increased the percentage of MYOD^+^ cells normalized to total or proliferating cells (Supplemental Figure 1, I and J), indicating that PN induces differentiation independently of proliferation. To understand the molecular basis of each molecule, we analyzed the transcriptome of vehicle-, NAM-, PN-, and NAM/PN combination–treated hMPs (Figure 1, F–I; Supplemental Figure 1, K–M; and Supplemental Tables 3 and 4). Gene set enrichment analysis revealed upregulation of pathways involved in cell cycle progression and cell division following treatment with NAM (Figure 1F), as expected from the increase in the number of proliferating progenitors (Figure 1E and Supplemental Figure 1E). PN-treated hMPs had a strong signature of protein synthesis (Figure 1G), consistent with myogenic differentiation requiring increased protein translation (32). While both NAM and PN increased the number of MYOD^+^ hMPs, transcriptomic and phenotypic analyses suggested that both molecules modulate different, complementary myogenic functions in vitro. When combining NAM and PN treatments, the individual transcriptional signature of each molecule was conserved (Figure 1, F–H, and Supplemental Figure 1, K–M). Notably, 630 genes were differentially upregulated by the NAM/PN combination over single treatments (Figure 1I and Supplemental Table 4), highlighting that NAM and PN synergize to regulate hMP gene expression. At the phenotypic level, compared with hMPs treated with NAM or PN alone, the NAM/PN combination synergistically increased the number of MYOD^+^ cells and improved both the EC_50_ and the maximal efficacy over the effects of single treatments (Figure 1J). NAM/PN also maintained the ability to stimulate PAX7^+^ hMP proliferation (Figure 1K) and enhanced terminal differentiation and myotube maturation (Figure 1, L–O, and Supplemental Figure 1, N–P). The effects of NAM/PN combination on differentiation resulted from early-stage hMP amplification and were not observed when the NAM/PN combination was supplemented after myotube formation was induced (Supplemental Figure 1, Q–U). An important feature of MuSC fate is the capacity to concomitantly engage in myogenic differentiation while self-renewing a pool of cells that maintains stemness. This process can be modeled in vitro by assessing the capacity of myoblasts to form PAX7^+^ reserve cells via niche signals derived from myotubes during differentiation (33). To test if the NAM/PN combination regulates self-renewal independent of its effect on MuSC proliferation and differentiation, we analyzed reserve cells in hMPs treated with NAM/PN after the induction of myotube differentiation. The effects of NAM/PN combination on proliferation and differentiation (Figure 1, C–E) did not come at the expense of self-renewal as it actually increased the number of PAX7^+^ reserve cells after differentiation (Figure 1P). We also tested if the NAM/PN combination cross-talks with cell proliferation of other human cells with high proliferation rates. The effects on hMP amplification were specific to myogenic progenitors and not recapitulated in nonmyogenic cells, as the NAM/PN combination did not stimulate proliferation of skin fibroblasts, unlike the positive control FGF (Supplemental Figure 1, V and W). Altogether, our in vitro results demonstrate that NAM and PN can be combined to stimulate myogenesis by modulating both myogenic progenitor proliferation and differentiation specifically.

### In vivo NAM/PN treatment stimulates MuSCs, accelerates regeneration, and increases muscle strength after muscle injury.

**Figure 2:**
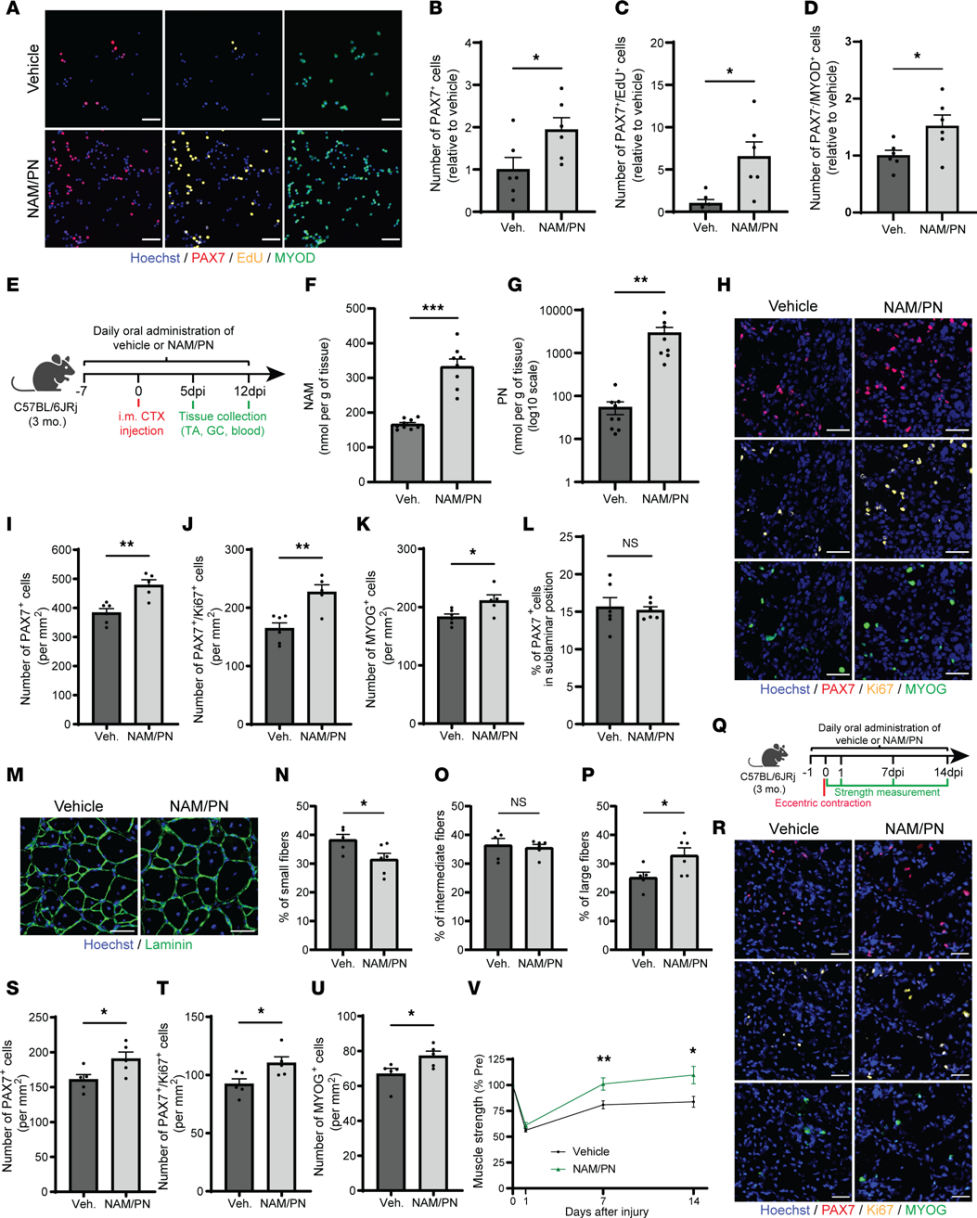
The combination of NAM and PN enhances MuSC function in vivo and increases muscle strength during regeneration. (**A**–**D**) Representative immunofluorescence images and quantification of FACS-isolated mouse MuSCs treated with vehicle or NAM/PN combination ex vivo for 4 days. *n* = 6 cell culture replicates with cells pooled from *N* = 4 mice. (**E**) Cardiotoxin-induced muscle regeneration in young mice treated orally with NAM/PN combination or vehicle. (**F** and **G**) NAM and PN concentrations quantified by LC-MS/MS in uninjured gastrocnemius (GC) muscles from young vehicle- (*N* = 9) and NAM/PN combination–treated (*N* = 8) mice. (**H**–**K**) Representative immunofluorescence images (**H**) and quantification of PAX7^+^ (**I**), PAX7^+^Ki67^+^ (**J**), and MYOGENIN^+^ (**K**) cells in tibialis anterior (TA) cross-sections from vehicle- (*N* = 6) and NAM/PN combination–treated (*N* = 5) mice at 5 dpi. (**L**) Number of PAX7^+^ sublaminar MuSCs in TA cross-sections from vehicle- (*N* = 6) and NAM/PN combination–treated (*N* = 6) mice at 12 dpi. (**M**–**P**) Representative immunofluorescence images (**M**) and quantification of minimum ferret of small (**≤**33 μm) (**N**), intermediate (>33 μm and **≤**43 μm) (**O**), and large (>43 μm) (**P**) regenerating myofibers in TA cross-sections from vehicle- (*N* = 5) and NAM/PN combination–treated (*N* = 6) mice at 12 dpi. (**Q**) Eccentric contraction–induced (EC-induced) muscle regeneration after electrically evoked lengthening contractions of plantar flexor (PF) muscles in young vehicle- and NAM/PN combination–treated mice. (**R–U**) Representative immunofluorescence (**R**) and quantification of PAX7^+^ (**S**), Ki67^+^ (**T**), and MYOGENIN^+^ (**U**) cells in GC muscle from vehicle- (*N* = 5) and NAM/PN combination–treated (*N* = 5) mice 7 days after the EC protocol. (**V**) Quantification of muscle strength (single twitch peak torque) in PF muscles before and 1, 7, and 14 days after EC-induced injury. *N* = 12 mice. Data are shown as the mean ± SEM. *P < 0.05; **P < 0.01; ***P < 0.001 with 2-tailed unpaired Student’s *t* tests (**B**–**D**, **F**, **G**, **I**–**L**, **N**–**P**, and **S**–**V**). Scale bars: 50 μm.

To evaluate if NAM/PN combination can support MuSCs in a complex physiological system and across species, lineage^–^ (CD31^–^CD45^–^CD11B^–^SCA1^–^) and CD34^+^ITGA7^+^ MuSCs were isolated from mouse hindlimb muscles by FACS and treated ex vivo with NAM/PN. The NAM/PN combination increased the total number of PAX7^+^ as well as PAX7^+^EdU^+^ proliferating MuSCs (Figure 2, A–C) and stimulated their progression toward MYOD^+^ progenitors (Figure 2, A and D). To substantiate these results in vivo, we analyzed MuSC function in a preclinical model of muscle regeneration after an acute muscle injury induced with cardiotoxin (CTX) (Figure 2E). Following oral administration, NAM and PN were well absorbed and bioavailable in skeletal muscle, as both their circulating (Supplemental Figure 2A) and intramuscular levels (Figure 2, F and G) were strongly increased compared with those in the vehicle-treated group. NAM/PN treatment was also well tolerated with no sign of toxicity (Supplemental Figure 2, B–D).

NAM/PN supplementation significantly increased by 38% the number of PAX7^+^ MuSCs and the number of proliferative PAX7^+^ cells measured via Ki67 at 5 days after injury (dpi) (Figure 2, H–J). Consistent with the effects of NAM and PN on early markers of differentiation in cell culture (Figure 1, C and D, and Figure 2, A and D), the NAM/PN combination also increased the number of differentiating MYOGENIN^+^ progenitors in vivo (Figure 2, H and K). To ensure that NAM/PN did not perturb MuSC self-renewal in vivo, we quantified return to quiescence once the majority of differentiation and self-renewal decisions had occurred. The NAM/PN combination did not affect return to quiescence, as the number of noncycling sublaminar PAX7^+^ MuSCs in regenerating areas did not differ between vehicle- and NAM/PN combination–treated muscle at 12 dpi (Figure 2L). The acceleration of myogenic repair translated into increased regenerating myofiber size at 12 dpi, with a 6.2% shift toward larger more mature fibers (Figure 2, M–P, and Supplemental Figure 2E). Altogether, these results indicate that the NAM/PN combination enhances muscle regeneration in vivo in young healthy conditions by accelerating MuSC dynamics and fiber repair without compromising self-renewal.

To investigate the functional relevance of the NAM/PN combination during muscle recovery, we measured muscle strength in a physiological mouse model of skeletal muscle regeneration induced by eccentric exercise using a standardized electrically evoked lengthening contraction protocol (Figure 2Q) (34). Consistent with the results in the severe model of CTX-induced muscle regeneration (Figure 2, H–K), the NAM/PN combination also increased the proliferation of PAX7^+^ MuSCs and their myogenic differentiation to MYOGENIN^+^ progenitors in this milder model of contraction-induced regeneration (Figure 2, R–U). Muscle strength was recorded longitudinally using a noninvasive set-up where evoked contraction recorded on a foot pedal was measured 1 day after injury, during the dynamic myofiber remodeling phase at 7 dpi and during the fiber maturation process at 14 dpi (Figure 2Q). Following eccentric contraction-induced injury, muscle strength dropped by 50% and gradually recovered across the 14-day time course (Figure 2V). Muscle strength was 27% higher in NAM/PN combination–treated compared with vehicle-treated animals at 7 dpi, and this difference was maintained at 14 dpi, enabling a more rapid return to preinjury strength levels (Figure 2V). These results across independent models of regeneration demonstrate that the NAM/PN combination supports all phases of muscle repair by boosting the initial amplification of MuSCs and inducing their progression toward differentiation, resulting in better muscle strength throughout the entire recovery process.

### NAM and PN signal through selective CK1-mediated β-catenin activation independent of NAD^+^ metabolism and AKT signaling, respectively.

**Figure 3:**
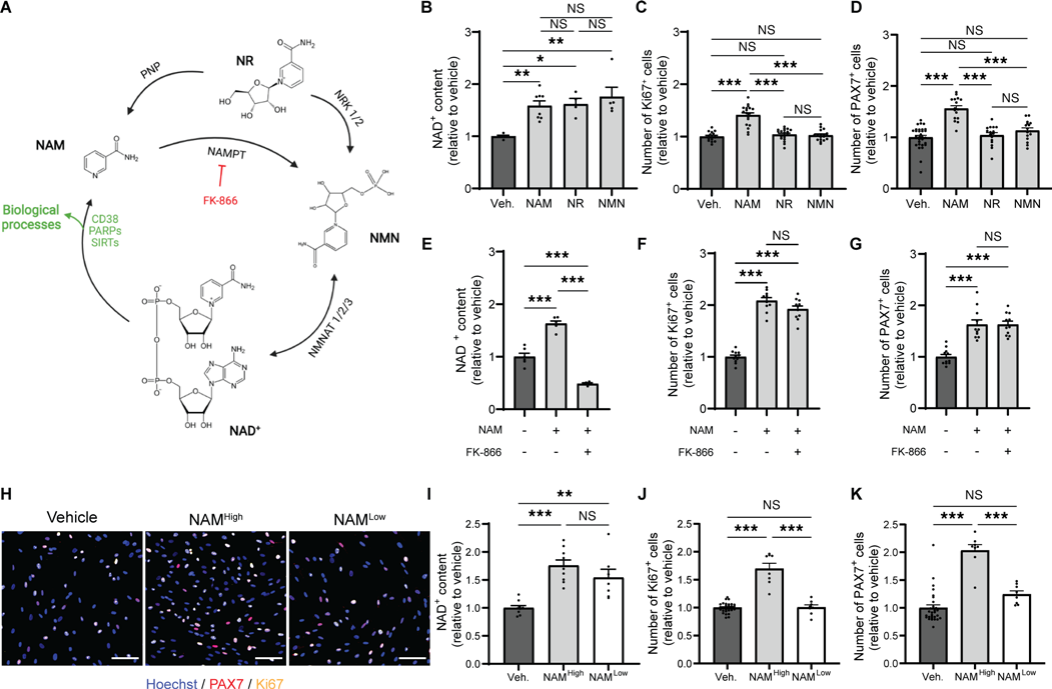
Nicotinamide promotes human myogenic progenitor proliferation independently of NAD^+^ metabolism. (**A**) Scheme of mammalian NAD^+^ biosynthesis from various NAD^+^ precursors. NAD^+^, nicotinamide adenine dinucleotide; NR, nicotinamide riboside; NMN, nicotinamide mononucleotide; NAMPT, nicotinamide phosphoribosyl transferase; NMNAT, nicotinamide mononucleotide adenylyl transferase; NRK, nicotinamide riboside kinase; PNP, purine nucleoside phosphorylase. (**B**–**D**) NAD^+^ content (**B**) and number of Ki67^+^ (**C**) and PAX7^+^ (**D**) human myogenic progenitors (hMPs) after treatment with vehicle or different NAD^+^ precursors. *n* ≥ 4 and *n* ≥ 15 cell culture replicates per condition from *N* = 2 donors for **B** and for **C** and **D**, respectively. (**E**–**G**) NAD^+^ content (**E**) and number of Ki67^+^ (**F**) and PAX7^+^ (**G**) hMPs after treatment with NAM and the NAMPT inhibitor FK-866. *n* ≥ 4 and *n* ≥ 11 cell culture replicates per condition from *N* = 2 donors for **E** and for **F** and **G**, respectively. (**H**–**K**) Representative images (**H**), NAD^+^ levels (**I**), and number of Ki67^+^ (**J**) and PAX7^+^ (**K**) hMPs after treatment with NAM at low (100 μM) and high (1 mM) doses. Scale bar: 100 μm. *n* ≥ 7 (**I**) and *n* ≥ 8 (**J** and **K**) cell culture replicates from 1 donor. Data are shown as the mean ± SEM. *P < 0.05; **P < 0.01; ***P < 0.001 with 1-way ANOVA with post hoc Tukey’s multiple comparison test (**b**–**G** and **i**–**K**).

Given the role of NAM as an NAD^+^ precursor and the importance of NAD metabolism in skeletal muscle homeostasis and regeneration (24, 35), we examined the potential of increasing endogenous NAD^+^ levels to stimulate MuSCs. Treatment of hMPs with the NAD^+^ precursors NAM, NAM riboside (NR), and NAM mononucleotide (NMN) (Figure 3A) resulted in comparable increases of NAD^+^ levels (Figure 3B). However, only NAM increased the number of PAX7^+^ and Ki67^+^ hMPs (Figure 3, C and D). To confirm these in vitro findings, we compared the effects of NAM and NR on muscle regeneration in vivo and evaluated whether boosting NAD^+^ levels in healthy muscle was sufficient to enhance MuSC function. Supplementation with NR and NAM increased the NAD^+^ content in muscle to a similar extent (Supplemental Figure 3A), but only NAM increased the number of PAX7^+^, Ki67^+^, and MYOGENIN^+^ progenitors (Figure 2, H–K, and Supplemental Figure 3, B–E), suggesting that the amplification of myogenic progenitors is NAM specific. Since NR has been reported to convert to NAM via the enzyme Pnp in mammals (36, 37), we assessed NAM levels following NR supplementation in mice (Supplemental Figure 3, F and G). As expected, circulating levels of NAM increased following NR administration, but the circulating levels of NAM resulting from NR conversion were 50 times lower compared with the levels reached after NAM/PN supplementation (Supplemental Figure 3F). Unlike oral NAM/PN, NAM conversion following oral NR was not sufficient to increase NAM bioavailability in skeletal muscle (Supplemental Figure 3G). To further validate that NAM promotes myogenic cell proliferation independently of NAD^+^, we blocked the conversion of NAM to NAD^+^ through inhibition of NAMPT with FK-866. As expected, FK-866 inhibited the conversion of NAM to NAD^+^ (Figure 3E) but did not abolish the expansion of PAX7^+^ and Ki67^+^ hMPs by NAM (Figure 3, F and G). In addition to its role as an NAD^+^ precursor, NAM has previously been shown to signal via NAD-independent mechanisms at high concentrations (38). We therefore examined the effect of different doses of NAM on hMP proliferation and NAD^+^ biosynthesis (Figure 3, H–K). While both concentrations of NAM triggered a comparable increase in NAD^+^ levels (Figure 3I), only high concentrations of NAM enhanced hMP proliferation (Figure 3, H, J, and K). Collectively, our in vitro and in vivo data demonstrate that increasing NAD^+^ alone is not sufficient to improve the function of young healthy MuSCs and suggest that NAM can activate MuSCs independently of NAD^+^.

**Figure 4:**
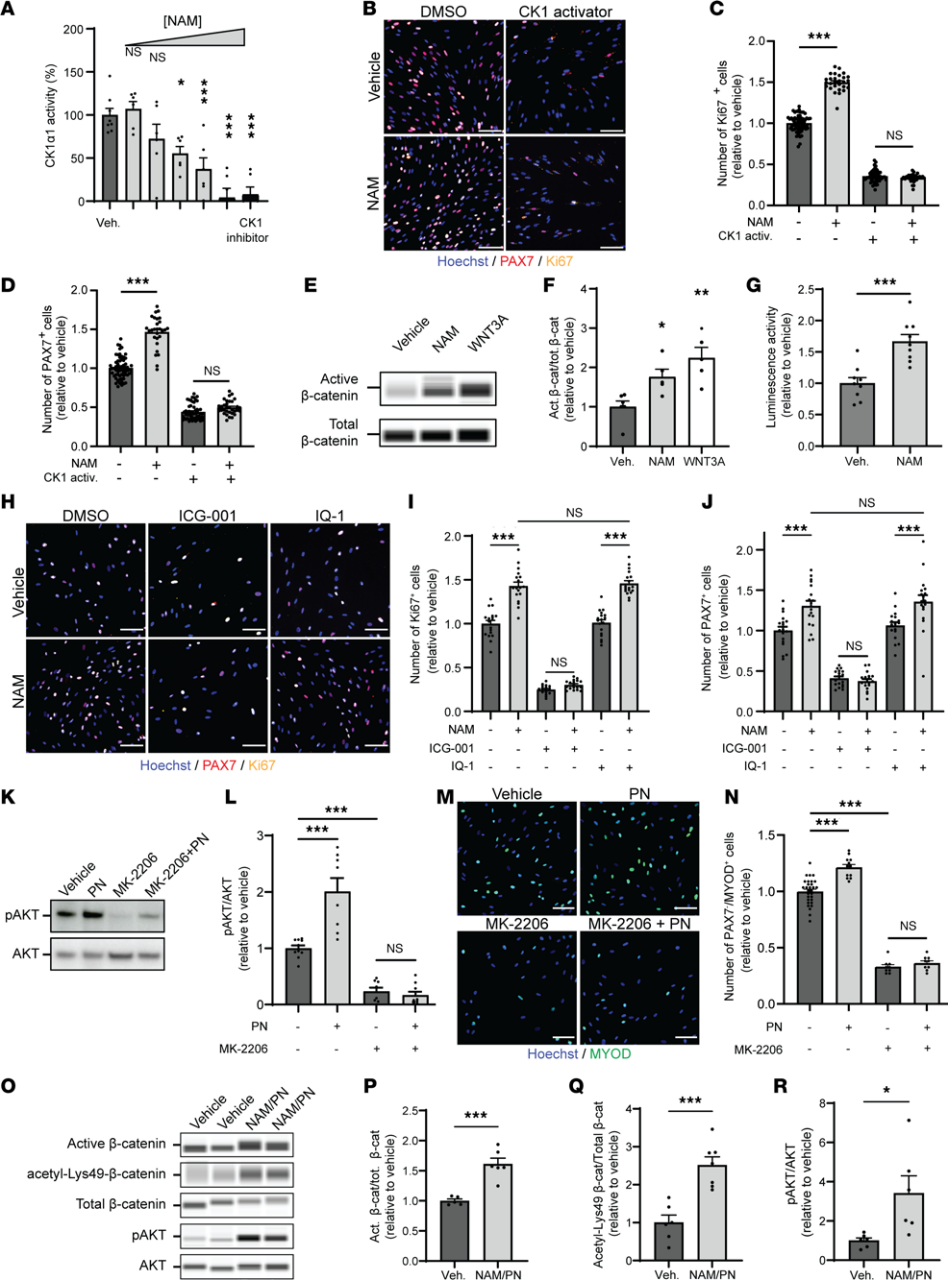
NAM and PN stimulate hMPs through β-catenin and AKT signaling, respectively. (**A**) Dose response of NAM on CK1α1 activity (1 μM to 20 mM). *n* ≥ 6 replicates. (**B**–**D**) Representative immunofluorescence images (**B**) and quantification of Ki67^+^ (**C**) and PAX7^+^ (**D**) hMPs after treatment with NAM and/or CK1α activator. *n* ≥ 16 cell culture replicates from 1 donor. (**E** and **F**) Representative capillary immunoassays (**E**) and quantification (**F**) of nonphosphorylated β-catenin protein levels in NAM-treated hMPs. WNT3A was used as positive control of β-catenin activation. *n* ≥ 5 cell culture replicates from *N* = 2 donors. (**G**) Luciferase activity of primary mouse MuSCs cotransfected with a TopFlash β-catenin luciferase reporter gene and treated with vehicle or NAM. *n* = 9 cell culture replicates. (**H**–**J**) Representative immunofluorescence images (**H**) and quantification of Ki67^+^ (**I**) and PAX7^+^ (**J**) hMPs after treatment with NAM and/or the β-catenin nuclear inhibitors ICG-001 and IQ-1. *n* ≥ 18 cell culture replicates from 1 donor. (**K** and **L**) Representative immunoblot images (**K**) and quantification (**L**) of hMPs treated with PN and/or the AKT inhibitor MK-2206. *n* ≥ 8 cell culture replicates from 1 donor. (**M** and **N**) Representative immunofluorescence images (**M**) and quantification of MYOD^+^ hMPs (**N**) following treatment with PN and/or MK-2206. *n* ≥ 9 cell culture replicates from 1 donor. (**O**–**R**) Representative capillary immunoassays (**O**) and quantification of active nonphosphorylated β-catenin protein levels (**P**), Lys49 acetylation of β-catenin (**Q**), and pAKT/AKT ratio (**R**) in MuSCs from regenerating muscles following oral NAM and PN supplementation in young mice. *N* ≥ 5 mice for each condition. Mouse MuSCs were freshly isolated from regenerating mouse TA, GC, and QD muscles at 5 dpi. Data are shown as the mean ± SEM. *P < 0.05; **P < 0.01; ***P < 0.001 with 1-way ANOVA with post hoc Dunnett’s (**A** and **F**), Šidák’s multiple comparisons adjustment (**C**, **D**, **I**, **J**, **L**, and **N**), or 2-tailed unpaired Student’s *t* test (**G**, **P**, **Q**, and **R**). Scale bars: 100 μm.

NAM inhibits casein kinase 1 α (CK1α) activity (Figure 4A) (38) at doses stimulating myogenic cell proliferation (Figure 1D and Figure 4A). Hyperactivation of CK1 drastically decreased the number of proliferating hMPs and was dominant over NAM, while both NAM and the pharmacological CK1 inhibitor increased hMP proliferation (Figure 4, B–D). These results demonstrate that CK1 is a regulator of MuSC proliferation and that NAM signals in hMPs via CK1 inhibition.

In nonmyogenic cells, CK1α directly phosphorylates cytoplasmic β-catenin to inhibit its activity by inducing proteosomal-mediated degradation (39). CK1 can also inhibit β-catenin in hMPs, as pharmacological CK1 hyperactivation increased phosphorylated inactive β-catenin (Supplemental Figure 4, A and B). Similar to NAM, pharmacological CK1 inhibition increased the number of proliferating Ki67^+^ and PAX7^+^ hMPs (Supplemental Figure 4, C and D). In line with its inhibition of CK1 (Figure 4A), NAM also increased active β-catenin 1.6-fold in hMPs to a smaller extent than WNT3A (Figure 4, E and F). To directly measure β-catenin transcriptional activity in MuSCs, we performed a luciferase reporter assay on freshly isolated MuSCs cotransfected with the TopFlash luciferase β-catenin reporter as previously described (40). 72 hours of NAM treatment increased β-catenin transcriptional activity by 66% (Figure 4G), demonstrating that the NAM-driven accumulation of β-catenin stimulates β-catenin dependent target gene activation.

Following translocation to the nucleus, active β-catenin interacts with two acetyltransferases, cAMP response element binding protein–binding protein (CBP) and E1A-binding protein, 300 kDa (p300), to drive the expression of its target genes (41). To better understand how NAM modulates β-catenin signaling, hMPs were treated with ICG-001 and IQ-1, two β-catenin antagonists that prevent the transcription of sets of β-catenin–dependent genes by disrupting the interaction between β-catenin and its coactivators CBP or p300, respectively (42, 43). Inhibition of the β-catenin/p300 interaction with IQ-1 did not impact basal or NAM-induced proliferation of hMPs (Figure 4, H–J). In contrast, inhibition of the β-catenin/CBP interaction with ICG-001 strongly decreased hMP proliferation and completely blocked the ability of NAM to stimulate hMP proliferation (Figure 4, H–J) without affecting cell viability or the ability to respond to the TGF-β inhibitor used as positive control (Supplemental Figure 4E). As an additional control, we verified that neither ICG-001 nor IQ-1 affected the capacity of PN to upregulate MYOD in hMPs (Supplemental Figure 4, F and G). These results suggest that NAM signals by activating a set of β-catenin target genes that is specifically regulated by the association with its coactivator CBP and demonstrate that NAM induces myogenic cell proliferation independent of NAD^+^ via a partial activation of β-catenin signaling via cytoplasmic CK1-mediated inhibition.

The AKT serine/threonine kinase controls myogenic differentiation and is a central regulator of anabolic processes and muscle protein synthesis (44), which were regulated in hMPs in response to PN (Figure 1G). Consistent with these transcriptomic profiles, PN treatment in hMPs increased the expression of the active phosphorylated form of AKT (Figure 4, K and L). AKT inhibition using the allosteric inhibitor MK-2206 decreased the number of MYOD^+^ cells (Figure 4, M and N). Importantly, the effects of PN were fully dependent on AKT, as AKT and PI3K inhibitors completely abolished the PN-induced upregulation of MYOD^+^ cells (Figure 4, M and N, and Supplemental Figure 4H), while maintaining sensitivity to the TGF-β inhibitor as positive control for myogenic induction (Supplemental Figure 4H). Moreover, treatment with an AKT activator mimicked the effect of PN on MYOD in hMPs (Supplemental Figure 4, I–K). As a control, we also verified that AKT inhibition by MK-2206 did not compromise the effect of NAM on hMP proliferation (Supplemental Figure 4, L–N).

Finally, to confirm that the molecular mechanisms of NAM and PN were conserved in vivo, we isolated activated MuSCs from regenerating muscles 5 dpi with and without oral NAM/PN supplementation and analyzed them by quantitative capillary Western Blot (Figure 4, O–R). Consistent with what we previously observed in hMPs, active β-catenin also accumulated in activated MuSCs from NAM/PN combination–treated mice (Figure 4, O and P). Moreover, the acetylation of lysine 49 on β-catenin, which is a readout of CBP-mediated activation of β-catenin (45), was also higher in NAM/PN combination–treated MuSCs (Figure 4, O and Q). Finally, AKT signaling was also upregulated in freshly isolated MuSCs following NAM/PN supplementation in vivo (Figure 4, O and R). Altogether, these molecular studies demonstrate that NAM and PN synergize to regulate MuSC proliferation and differentiation in hMPs and MuSCs in vivo through partial CK1-dependent β-catenin activation by NAM independently of NAD^+^ and AKT activation by PN.

### NAM/PN supplementation reverses MuSC dysfunction and regenerative decline during aging.

**Figure 5:**
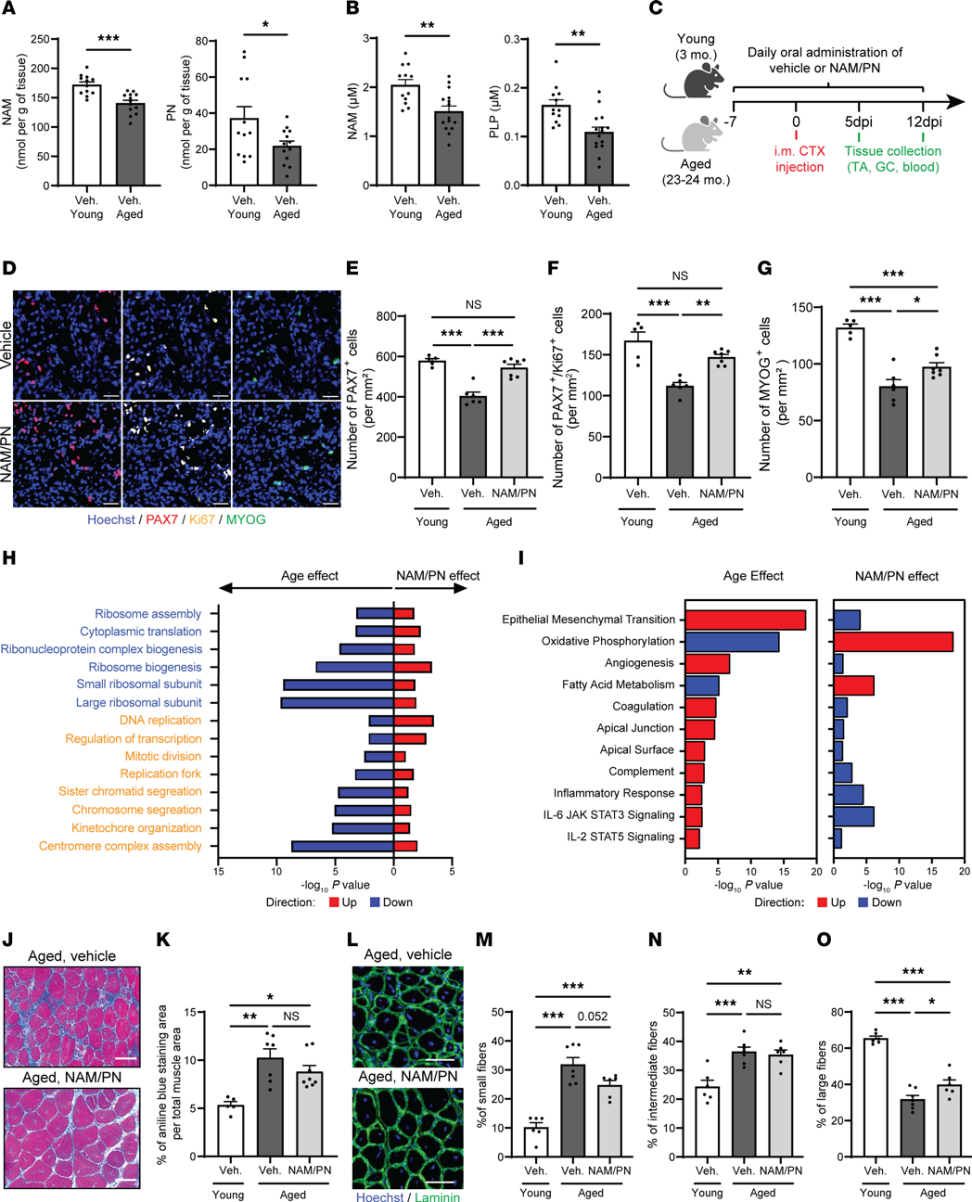
NAM and PN restore MuSC function and enhance regeneration in aged skeletal muscle. (**A** and **B**) Gastrocnemius (**A**) and plasma (**B**) concentrations of NAM and PN or NAM and PLP by LC-MS/MS in young and aged mice. *N* ≥ 12 mice per group.(**C**) Experimental scheme of CTX-induced muscle regeneration in young and aged mice treated with The NAM/PN combination or vehicle. (**D**–**G**) Representative immunofluorescence images (**D**) and quantification of PAX7^+^ (**E**), PAX7^+^Ki67^+^ (**F**), and MYOGENIN^+^ (**G**) cells on tibialis anterior (TA) cross-sections from young (*N* = 5) and aged vehicle- (*N* = 6) and NAM/PN combination–treated (*N* = 7) mice at 5 dpi. (**H**) Gene set enrichment analysis curated from Gene Ontology:Biological Process (GO:BP) gene sets (https://www.gsea-msigdb.org/gsea/msigdb) of freshly isolated MuSCs from young and aged mice (age effect) and of aged MuSCs treated ex vivo with vehicle or the NAM/PN combination (treatment effect) (*N* = 6). (**I**) Gene set enrichment analysis of curated Hallmarks gene sets of regenerating GC muscles of young vs. aged mice and of vehicle- vs. NAM/PN combination–treated aged mice 5 dpi (*N* = 6). False discovery rate, 10%. (**J** and **K**) Representative images (**J**) and quantification (**K**) of fibrotic aniline blue^+^ area from a Masson’s trichrome staining of TA cross-sections from young (*N* = 5) and aged vehicle- (*N* = 7) and NAM/PN combination–treated (*N* = 8) mice at 12 dpi. (**L**–**O**) Representative immunofluorescence images (**L**), quantification of minimum ferret myofiber size of small (**≤**22 μm) (**M**), intermediate (>22 μm and **≤**32 μm) (**N**), and large (>32 μm) (**O**) regenerating myofibers in TA cross-sections from young (*N* = 6) and aged vehicle- (*N* = 7) and NAM/PN combination–treated (*N* = 6) mice at 12 dpi. Data are shown as the mean ± SEM. *P < 0.05; **P < 0.01; ***P < 0.001 with 2-tailed unpaired Student’s *t* test (**A** and **B**) and 1-way ANOVA followed by post hoc Tukey’s (**E**–**G**, **K**, and **M**–**O**) multiple comparison tests. Scale bars: 50 μm.

Since aging causes functional decline of MuSCs and defective regeneration, we next evaluated whether endogenous NAM/PN levels are altered during aging and if supplementation of the NAM/PN combination can rescue MuSC function and ameliorate muscle repair in aged mice. NAM and PN levels declined spontaneously during aging with muscle concentrations reduced in aged compared with young mice by 18% and 37%, respectively (Figure 5A). A comparable 25%–35% age-related decrease of NAM and pyridoxal-5′-phosphate (PLP), the bioactive form of PN, was also observed in plasma (Figure 5B), highlighting that aging causes a global systemic decline of NAM and PN metabolism. Oral NAM/PN supplementation could overcome this age-related deficit (Supplemental Figure 5, A and B), demonstrating that the intestinal absorption of NAM and PN is not impaired during aging.

After CTX-induced muscle injury in aged mice (Figure 5C), the NAM/PN combination treatment counteracted age-related impairments of MuSCs. The number of PAX7^+^ MuSCs decreased by 43% in aged muscle at 5 dpi and was fully rescued by NAM/PN combination treatment (Figure 5, D and E). PAX7^+^ cell amplification was driven by a regulation of MuSC proliferation as the number of PAX7^+^Ki67^+^ cells decreased significantly during aging and was rescued by NAM/PN (Figure 5, D and F). The NAM/PN combination also increased the number of MYOGENIN^+^ cells at 5 dpi in aged muscle, thereby mitigating the age-related defects in myogenic progenitor differentiation (Figure 5, D and G). We next evaluated the transcriptional changes induced by the NAM/PN combination in MuSCs freshly isolated from aged mice or in aged regenerating muscle from NAM/PN combination–treated mice. When compared with young MuSCs, pathways involved in MuSC proliferation and differentiation were downregulated in aged MuSCs and reactivated in aged MuSCs treated with NAM/PN (Figure 5H). Aging disrupts molecular signatures involved in inflammation, metabolism, and muscle repair in regenerative muscle (46), and these alterations were reversed by treatment with the NAM/PN combination (Figure 5I). Importantly, NAM/PN had minimal effect on the transcriptional landscape of uninjured muscle and did not significantly reverse aging signatures of uninjured muscle (Supplemental Figure 5C), suggesting that the NAM/PN combination primarily cross-talks with repair mechanisms in regenerating muscle. Gene expression signatures also suggested that NAM/PN treatment could reduce the expression of profibrotic genes in aged regenerating muscle (Supplemental Figure 5D). However, acute NAM/PN treatment during regeneration did not affect fibrosis, as measured by aniline blue staining, in regenerating or uninjured muscles (Figure 5, J and K, and Supplemental Figure 5, E and F), suggesting that the NAM/PN combination exerts its beneficial effects primarily by enhancing MuSC function and may fine-tune extracellular matrix remodeling without acute functional consequences on fibrosis. The enhanced MuSC function in aged muscle with NAM/PN translated into enlarged regenerating myofibers with centralized nuclei at 12 dpi (Figure 5, L–O), with a 25% increase in the number of larger, more mature fibers in NAM/PN combination–treated mice that translated to an overall increase in the myofiber area distribution of 7.6% (Figure 5O and Supplemental Figure 5G). No fiber size differences were observed between vehicle- and NAM/PN combination–treated mice in uninjured contralateral muscles (Supplemental Figure 5, H–J). Our molecular and histological results thus demonstrate that NAM/PN combination reverses the aging phenotype of MuSCs and mitigates muscle aging specifically in conditions where MuSCs are active.

### NAM and PN are reduced in older people with impaired physical function and reverse human age-related myogenic decline.

**Figure 6:**
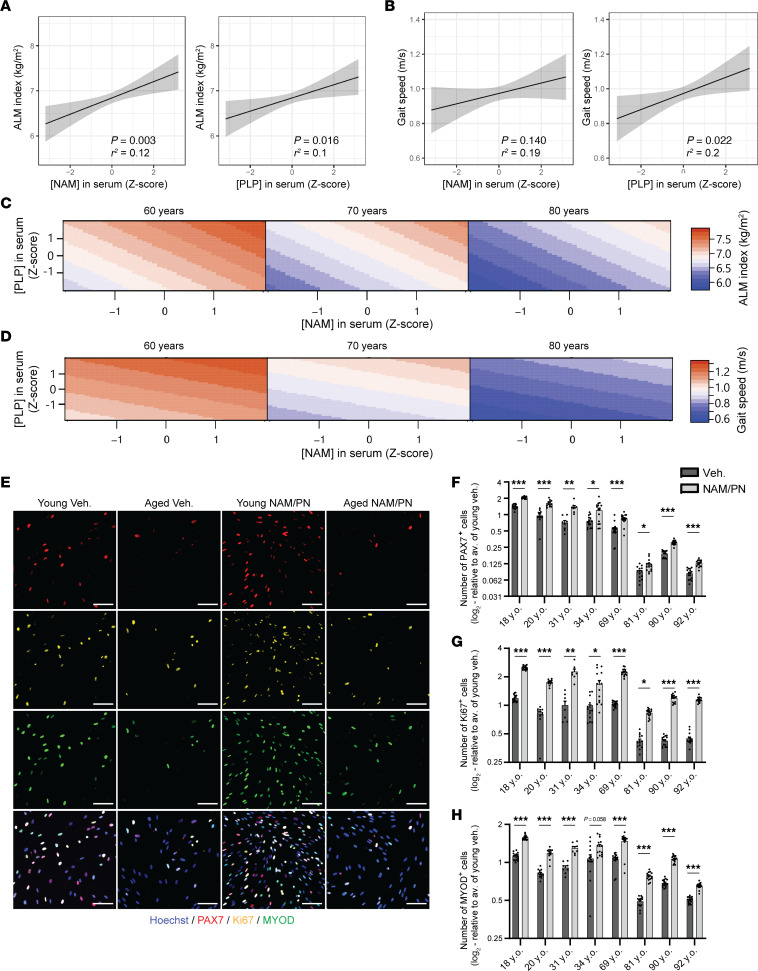
Nicotinamide and pyridoxine associate with muscle mass and function in aged humans and restore the myogenic capacity of aged hMPs. (**A**–**D**) LC-MS/MS analyses of NAM and pyridoxal-5′-phosphate (PLP; bioactive pyridoxine) in the sera of men aged >60 years (*N* = 186 participants). Appendicular lean mass index (ALMi) (**A**) and gait speed (**B**) were correlated to serum concentrations of NAM and PLP using a linear regression model adjusted for age. Regression line (black) and 95% confidence interval (CI) (gray). Estimated outcomes of the combined effect of NAM and PLP on ALMi (**C**) and gait speed (**D**) were modeled at different ages using a multiple linear regression model adjusted for age. (**E**–**H**) Representative immunofluorescence images (**E**) and quantification of PAX7^+^ (**F**), Ki67^+^ (**G**), and MYOD^+^ (**H**) hMPs. *n* ≥ 8 cell culture replicates from *N* = 8 donors aged from 18 to 92 years following 72 hours of treatment with vehicle or the NAM/PN combination. Data are shown as the mean ± SEM. **P < 0.01; ***P < 0.001 with Brown-Forsythe and Welch’s ANOVA tests with Dunnett’s T3 multiple-comparison in 8 donors (**F**–**H**). Data are shown as the mean ± SEM. Scale bar: 100 μm.

To investigate the relevance of NAM and PN for human muscle physiology during aging, concentrations of NAM and PLP, the active form of PN, were measured in the sera of 186 randomly selected older men aged 60 years and above from the Bushehr Elderly Health (BEH) program (47) (Table 1). In this cohort, with a 46% prevalence of sarcopenia (Table 1), circulating concentrations of NAM and PLP were low in individuals with low muscle mass, as assessed via the appendicular lean mass index (ALMi) measured using a dual-energy X-ray absorptiometry scan, and significantly associated with ALMi across all individuals (Figure 6A and Supplemental Table 5). Similar associations were observed with total ALM (Supplemental Table 5), while normalization to BMI reduced these associations, likely because it introduced more variability in the assessment of lean mass. Circulating levels of other hydrosoluble vitamins with similar metabolism and physicochemical properties, such as thiamin (vitamin B1) and riboflavin (vitamin B2), were not associated with muscle mass measured via the ALMi (Supplemental Figure 6, A and B), demonstrating that the clinical association of NAM and PN with muscle mass is not an indirect consequence of lower capacity to store micronutrients. Gait speed, a validated clinical variable to assess muscle function that predicts quality of life and mortality (48), was also positively associated with circulating levels of PLP and to a lesser extent of NAM (Figure 6B). We used statistical models to evaluate potential dietary causes of the associations as well as functional interdependencies of NAM, PLP, and age. ALMi and gait speed did not associate with dietary intake of vitamin B3 and B6 measured via food frequency questionnaires but remained significantly correlated with sera levels of NAM and PLP when the statistical models were corrected for dietary intake (Supplemental Table 6). A biostatistical model using multiple linear regression adjusted for age demonstrated that NAM and PLP levels directly associate with ALMi and gait speed in all decades analyzed from 60 to 80 years, regardless of the extent of functional decline with age (Figure 6, C and D). Importantly, the contributions of NAM and PLP were interdependent and additive at all ages tested, demonstrating that there is a functional interaction between the levels of NAM and PLP in humans to influence physical capacity.

Reduced myogenic capacity during aging is a hallmark of human sarcopenia (12, 46) and was recapitulated in hMPs from 4 young and 4 aged individuals (Figure 6, E–H; Supplemental Figure 6, C–E; and Supplemental Table 1). Treatment with the NAM/PN combination could counteract human age-related myogenic decline by increasing the proliferation of aged PAX7^+^ hMPs (Figure 6, E–G, and Supplemental Figure 6, F–I), their activation of MYOD (Figure 6H and Supplemental Figure 6J), and their terminal differentiation to myotubes (Supplemental Figure 6, K–N). The effect size of the NAM/PN combination on myogenic readouts was equivalent in all donors and ages tested (Supplemental Figure 6, H–J). Altogether, our results on clinical cohorts and primary human cells demonstrate that NAM and PN are clinically relevant nutrients that decline in human sarcopenia and reverse the myogenic defects of aged human myogenic cells.

## Discussion

We used a high-content imaging screen on hMPs and identified NAM and PN as nutrients that enhance MuSC proliferation and myogenic regeneration. NAM and PN have been recognized as safe by the FDA and EFSA (49, 50) and are approved for broad use in foods or dietary supplements. Targeting MuSCs with safe nutritional solutions is particularly relevant to accelerate physiological muscle recovery during MuSC-mediated repair of myofiber microdamage following exercise, sports-induced muscle tears and injuries, or following surgical procedures that mechanically rupture myofibers (5, 51). MuSCs are also impaired in genetic and chronic muscle diseases such as aging, dystrophies, cancer cachexia, or diabetes (52–54). The activation of MuSCs by NAM/PN combination, which enhances regeneration and accelerates the recovery of muscle strength, is therefore a potent therapeutic solution with potential broad applications in the management of healthy healing processes and exercise adaptations as well as prevention and nutritional management of muscle wasting disorders.

The efficacy of NAM/PN involves complementary molecular mechanisms that synergistically enhance proliferation and myogenic capacity of MuSCs. PN stimulates MuSC differentiation by accelerating MyoD activation via AKT signaling and protein synthesis. While stimulation of protein synthesis is important for MuSC differentiation (32), conversion of PN to its bioactive form PLP also catalyzes several rate-limiting reactions for energy metabolism (55), which may also contribute to myogenic fate directly or synergize with AKT signaling as also reported in other tissues and species (56–58). Further analyses will be important to understand if PN activates PI3K/AKT signaling via a specific molecular target or enhances AKT signaling and myogenic differentiation secondary to metabolic adaptations via PLP-sensitive enzymes. NAM is a precursor for the critical cellular cofactor NAD^+^ and is incorporated into NAD^+^ from dietary sources or intracellular metabolism via the salvage pathway (59). Our in vivo studies of healthy young mice showed that, unlike NR, which contributes to NAD^+^ homeostasis in conditions of NAD^+^ deficiency during aging or disease (24), stimulation of MuSC proliferation by NAM did not require conversion to NAD^+^. While NR has been shown to convert to NAM via hepatic metabolism (60) and this conversion could be detected in vivo during regeneration, the levels of NAM generated from NR were insufficient to stimulate healthy MuSC proliferation. In contrast, NAM stimulates MuSC proliferation via a selective activation of cytoplasmic β-catenin signaling via derepression of CK1α-mediated phosphorylation, which facilitates β-catenin acetylation and nuclear translocation. Similar to NAM, pharmacological modulation of CK1α modulated hMP proliferation, and treatment with a CK1α activator blocked the effects of NAM. Inhibition of β-catenin interaction with its coactivator CBP, but not p300, abrogated the effects of NAM on PAX7^+^ cells, highlighting that MuSCs are also sensitive to the NAM-dependent selective recruitment of coactivators to β-catenin observed in other lineages (61). Previous studies have suggested that the cross-talk of β-catenin signaling with myogenesis is complex. While activation of canonical Wnt signaling during skeletal muscle repair is well established (62, 63), the role of β-catenin in myogenesis is debated (62–66) and depends on diverse spatiotemporal signals across cellular compartments and cell types. Genetic deletion of β-catenin in MuSCs originally suggested that β-catenin is dispensable for regeneration (64). However, several subsequent studies using mice with constitutively active or knockout β-catenin have shown that β-catenin signaling can modulate MuSC function and impact regeneration (40, 62, 63, 65, 66). The different phenotypes reported upon β-catenin modulation with various tools highlight that this pathway integrates multiple inputs with different sensitivity and that permanent genetic deletion/overexpression of β-catenin leads to different molecular and phenotypic outcomes compared with pharmacological inhibition using Wnt ligands or secreted Frizzled-related proteins. While our findings suggest that a mild and transient activation of cytoplasmic β-catenin via CK1α inhibition and CBP recruitment is beneficial and supports MuSC proliferation, NAM treatment in genetic models where CK1 or β-catenin is specifically ablated in MuSCs or where specific β-catenin/CBP interaction sites are mutated will be important to confirm the molecular mechanisms through which NAM cross-talks with MuSC proliferation and improves regeneration.

In our study, levels of NAM and PN associated with muscle decline during aging, and NAM/PN treatment at therapeutic doses could overcome the detrimental effects of aging on MuSC exhaustion and regenerative decline (15). In addition to stimulating healthy MuSCs, NAM/PN treatment reversed the defective proliferation and MYOD activation of aged MuSCs and enhanced regeneration. NAM/PN also rescued the impaired proliferation and differentiation of hMPs from aged and geriatric humans, demonstrating that the myogenic-activating mechanisms of NAM/PN were dominant over the pathways that decline during aging in rodent and human muscle. Several lines of evidence support that the benefits of NAM/PN supplementation on age-related muscle decline cross-talk with myogenic repair mechanisms. Improved myofiber size and reversal of transcriptomic signatures of aging were specific to regenerating muscle but were not or were minimally affected in uninjured muscle. In addition, NAM/PN enhanced myogenic differentiation when myogenic progenitors were treated but did not affect terminal myotube maturation in vitro. While these results are consistent with a positive effect of NAM/PN on muscle regeneration via myogenic stimulation of MuSCs and their progeny, NAM/PN may also affect nonmyogenic cells of the niche that support muscle regeneration (16) and enhance repair mechanisms directly in myofibers (67). Further studies using MuSC-depleted mice will therefore be important to test whether NAM/PN may also support muscle health by stimulating complementary MuSC-independent mechanisms.

In our epidemiology study, low circulating levels of NAM and the bioactive form of PN associated with reduced muscle mass and gait speed in older people. Since muscle mass and gait speed are clinical variables used to define sarcopenia and established predictors of physical fitness, quality of life, and survival (12), our results suggest that endogenous inadequacy of these metabolites could contribute to loss of functional capacity during aging. Dietary intake does not seem to be the primary cause linking endogenous NAM/PN levels to muscle health, and the association of both nutrients in serum with muscle mass and function was interdependent, suggesting that altered endogenous metabolism of these nutrients during aging could explain their association with muscle phenotypes. Impaired regeneration and poor recovery from acute traumatic events, such as injuries, surgeries, or falls, is an important contributing mechanism to the progression of sarcopenia (12). Given the indispensable role of MuSCs in regeneration and recovery from myofiber injury, it is possible that low levels of NAM and PN contribute to sarcopenia via a regenerative mechanism through MuSCs. However, we could not collect muscle biopsies to quantify MuSCs in this clinical study and could therefore not relate clinical outcomes to MuSC activation. Since NAM and PN regulate general metabolic pathways across different cell types, it is possible that NAM and PN may associate with muscle mass and function via an effect in myofibers or other cell types. Collectively, our results support a translational application of NAM/PN in older people with physical decline, especially during the phases of acute recovery. However, sarcopenia has multifactorial origins, and it will be important to combine NAM/PN with physical activity and other nutrients such as protein, vitamin D, and omega 3 fatty acids, which are part of the standard of care to manage different physiological mechanisms that contribute to sarcopenia.

In summary, NAM/PN supplementation is an effective therapeutic strategy to stimulate MuSCs. Our work in preclinical models, primary human cells, and in an observational clinical cohort further establishes NAM/PN as a new translational solution to accelerate skeletal muscle repair and mitigate age-associated regenerative decline by targeting MuSC activation via regenerative nutrition.

## Methods

The detailed experimental procedures and reagents utilized in this study are described in the Supplemental Methods.

### Sex as a biological variable.

For human cells, hMPs from both male and female donors were used (Supplemental Table 1). All mouse experiments were performed using male mice to maintain consistency throughout the study, as aged female mice were not commercially available from the supplier. The nutritional observational study from the BEH cohort was analyzed in men to maximize statistical power, given gender differences in micronutrients during aging (68).

### Primary hMPs.

Primary hMPs (Lonza, CC-2580 or Cook Myosite, SK-111; Supplemental Table 1) were selected for myogenic purity (>90% desmin^+^ cells, CD56^+^) and absence of fibroblast contamination (<5% α smooth muscle actin^+^ cells). We also controlled all hMPs for their capacity to differentiate into myotubes with a fusion factor >50%, as assessed by myosin heavy chain or troponin T. For each experiment, a frozen stock of hMPs cells banked with fewer than 4 passages was thawed and expanded in Skeletal Muscle Cell Growth Medium (AmsBio, SKM-M) in a humidified incubator at 37°C in 5% CO_2_. hMPs were cultured for a maximum of 3 passages prior to cellular assays and were passaged upon reaching 50%–60% confluence, approximately every 3 days.

### High-throughput imaging phenotypic assay.

An automated high-throughput imaging phenotypic assay was developed to assess hMP proliferation in 384-well plates. All liquid dispensing, compound treatment and imaging steps were conducted on automated platforms optimized for this screen. A primary screen of 50,000 natural bioactive molecules and plant extracts from in-house libraries was performed on donor A using the percentage of PAX7^–^MYOD^+^ cells as primary readout. Assay conditions were optimized using the TGF-β inhibitor LY364947 (Sigma-Aldrich, L6293; ref. 69) as a positive control to reach an average Z′ factor above 0.5. Each 384-well plate contained 320 treatment conditions, each tested at 10 μM in 1% DMSO, 32 vehicle controls with 1% DMSO only, and 32 positive controls treated with 25 μM LY364947 in 1% DMSO. The percentage of PAX7^–^MYOD^+^ cells was normalized using the min-max scaling method, with “0” being attributed to the negative control and “1” to the positive controls, and the results were displayed and analyzed using Vortex software. All compounds classified by the FDA as GRAS were tested at 1 mM for primary screening and then were separately tested for confirmation in duplicates on hMPs from 2 donors. 800 cells were plated on 384-well plates precoated with 10 μg/mL human fibronectin (Corning, 356008) and grown in Skeletal Muscle Cell Growth Medium (AmsBio, SKM-M) for 72 hours under humidified conditions at 37°C in 5% CO_2_ before immunocytochemistry and image acquisition, performed as described in the Supplemental Methods.

### Secondary hMP proliferation and differentiation assays.

Primary cells were cultured as previously described (70), and experimental procedures and reagents used in this section are detailed in the Supplemental Methods. Secondary proliferation and differentiation assays were performed using hMPs from a total of 5 independent donors (Supplemental Table 1). Unless otherwise stated, the concentrations used for the different treatments were as follows: 1% DMSO as vehicle condition, 1 mM NAM (Enamine, EN300-15612), 1 mM PN (Enamine, EN300-39851), 1 mM NR (NR Chloride, ChromaDex, 00014332), 1 mM NMN (β-NAM mononucleotide, Sigma-Aldrich, N3501), 100 μM FK-866 (Sigma-Aldrich, F8557), 1 mM NAM (NAM^hi^), 100 μM NAM (NAM^lo^), 1 μM ICG-001 (R&D Systems, 4505/10), 1 μM IQ-1 (Sigma-Aldrich, 412400), 25 μM LY364947 (Sigma-Aldrich, L6293), 1 μM wortmannin (Sigma-Aldrich, W1628), 100 μM LY294002 (Sigma-Aldrich, L9908), 1 μM SC79 (Sigma-Aldrich, SML0749), 8 μM TAK-715 (Sigma-Aldrich, SML0360), 200 ng/mL WNT3A (R&D Systems, 5036-WN-010/CF), or 1 μM MK-2206 (Selleckchem, S1078). Immunocytochemistry and image acquisition were performed as described in the Supplemental Methods

### Other cellular assays.

Cellular assays with primary mouse cells and human primary fibroblasts are described in the Supplemental Methods.

### In vivo mouse experiments.

Mice were housed under standard conditions (up to 5 mice per cage) and allowed access to food and water ad libitum. Young (12–13 weeks old) and aged (23–25 months old) wild-type C57BL/6JRj male mice were purchased from Janvier labs. All mice were randomized to different groups according to their weights. Treatments resuspended in 1% sodium carboxymethyl cellulose (CMC) were administrated by daily oral gavage, starting prior to the injury and continuing until the endpoint of the study: NAM (200 mg/kg per day, Enamine, EN300-15612), PN (4 mg/kg day, Enamine, EN300-39851), or NAM riboside (NR, 200 mg/kg per day, ChromaDex, 00014332). Vehicle-treated mice received an equivalent volume of 1% CMC using the same dosing scheme every day (bolus of 200 μL maximum performed at the same time of the morning ±2 hours). For regeneration studies, muscle injury was induced by intramuscular injection of 20 μM CTX (Latoxan) into the tibialis anterior (25 μL) and the gastrocnemius (50 μL) muscles under anesthesia. For longitudinal muscle strength measurement, muscle injury was induced through eccentric contractions induced by electrical stimulations following a previously published protocol (34) that was adapted as described in the Supplemental Methods. Tissue harvesting and immunohistochemistry were performed as detailed in Supplementary Methods. Muscle sections were imaged using the VS120 and VS200 slide scanners (Olympus). Images were analyzed using the VS-ASW FL measurement tool and the QuPath software. For ex vivo assays, MuSCs were isolated with a Beckman Coulter Astrios Cell sorter as previously described (20). MuSC fate was assessed with PAX7 and MYOD immunostainings. Images were acquired using the ImageXpress (Molecular Devices) platform, and quantifications were performed using multiwavelength cell scoring.

### Human nutritional epidemiology study (BEH).

186 older men aged above 60 years and above were randomly selected from the BEH cohort for a metabolomic subanalysis (a detailed description of the entire cohort is available elsewhere; refs. 47, 71). The ALMi was used as an estimate of skeletal muscle mass using dual-energy X-ray absorptiometry and was calculated for each participant as the sum of upper and lower limb lean mass expressed in kilograms divided the height squared expressed in meters. Physical performance was evaluated using a 4.57 m gait speed test, with gait speed determined as the best of 2 repeats at the participant’s normal pace. An overnight fasting venous blood sample was collected by trained nurses for every participant, and sera were stored at –80°C before being analyzed as described in *Metabolomics analyses* in the Supplemental Methods.

### Molecular and biochemical assays.

Experiments were performed according to manufacturer’s protocols. All experimental procedures and reagents are described in the Supplemental Methods.

### Statistics.

Unless otherwise stated, data were analyzed using the Prism 9 software package and are represented as mean ± SEM. All analyses were performed using parametric statistics based on historical values of the lab. For all data, the significance threshold was set at *P* value less than 0.05. A 2-tailed Student’s *t* test was used to compare experimental conditions with only 2 groups, and 1-way ANOVA was used to compare experimental conditions with multiple groups using a Dunnett’s post hoc test to compare every group to a control and a Tukey’s post hoc or Holm-Šidák post hoc test to compare several groups. The Kolmogorov-Smirnov test was used to compare the cumulative distribution. Brown-Forsythe and Welch’s 1-way ANOVA test was used when groups do not have equal variances. For human serum analyses, concentrations of NAM and PLP were log_2_- and *Z* score–transformed (mean centering and dividing by the standard deviation), while age was *Z* score transformed. Multiple linear regressions adjusted for age were performed using R software to estimate the association between clinical response variables (ALM and gait speed) and *Z* scores of NAM, PLP, dietary intake, and age. Statistical analyses of RNA-sequencing experiments are described in the Supplemental Methods.

### Study approval.

Approval to use human cells for research purposes was obtained from the Vaud ethics commission for human research (CER–VD; Switzerland) under authorization PB_2016-00709. Animal experiments were approved by the veterinary office of the Canton of Vaud, Switzerland (authorization no. 3440 and 3690), and both the local ethic committee CEEA-55 and the French ministry of research (APAFIS, no. 30000-2021022210224394 v1). Humane termination endpoints had been established prior to the start of experimentation, as described in the animal authorizations. The BEH cohort consists of 3000 individuals aged over 60 years old and living around the city of Bushehr, Iran. A detailed description of the entire cohort is available elsewhere (47, 71). The study protocol was approved by the ethics committee of Endocrinology and Metabolism Research Institute, affiliated with Tehran University of Medical Sciences (Tehran, Iran) as well as the Research Ethics Committee of Bushehr University of Medical Sciences (Bushehr, Iran) under reference TUMS.EMRI.REC.1394.0036. Reanalyses performed in Switzerland were approved by the cantonal ethics commission for human research (CER-VD) in Vaud, Switzerland, under reference 490/14. Written informed consent was signed by all the participants.

### Data availability.

All software used was freely or commercially available. Any additional information required to reanalyze the data reported in this paper is available upon reasonable request. RNA-sequencing data have been deposited in GEO (accession GSE264284, GSE264285, GSE271744, and GSE269250). Other materials are available for sharing upon reasonable request within the limit of availability of nonrenewable samples. A Supporting Data Values file with all reported data values is available as part of the supplemental material. Complete unedited gel images are provided in the supplemental materials.

## Author contributions

SA, JM, PS, and JNF designed the experimental strategy, interpreted the results, and wrote the manuscript. SA, JM, SK, PG, SR, TD, LP, JLSG, and PS performed experiments and analyzed data. JM, SK, YR, BB, and DB developed and/or performed the cellular screen. CJ, AF, RM, and JG designed, performed, and analyzed the in vivo muscle contraction experiments. IS performed and analyzed β-catenin luciferase experiments. EM, AO, RH, FF, and JNF lead human metabolomics and analyzed/interpreted the results. SM and EM performed and analyzed transcriptomic experiments. GEJ, LT, and SM supported imaging, flow cytometry, and genomics. LGK and SB contributed to experimental strategy and data interpretation. PS and JNF conceived and lead the project. SA and JM are listed as co–first authors, as they shared primary responsibility in conducting experiments, analyses, and interpretation of results for this study. The order of authorship reflects the contribution to writing and editing of the manuscript. All authors read and approved the final manuscript.

## Supplementary Material

Supplemental data

Unedited blot and gel images

Supplemental table 1

Supplemental table 2

Supplemental table 3

Supplemental table 4

Supplemental table 5

Supplemental table 6

Supporting data values

## Figures and Tables

**Table 1:**
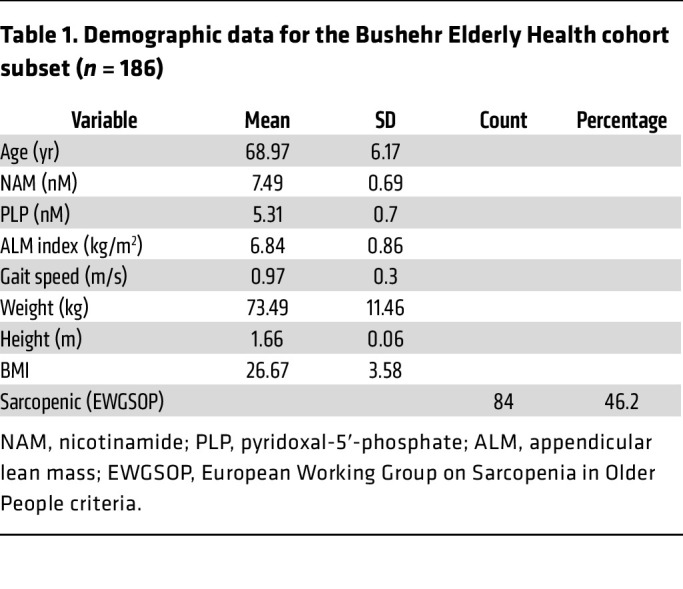
Demographic data for the Bushehr Elderly Health cohort subset (*n* = 186)

## Version 1 11/12/2024

In-Press Preview

## Version 2 12/16/2024

Electronic publication
