# Supplementary material for: Nicotinamide and pyridoxine stimulate muscle stem cell expansion and enhance regenerative capacity during aging
Source: J Clin Invest. 2024 Nov 12;134(24):e163648. doi: 10.1172/JCI163648 (PMC11645154; doi:10.1172/JCI163648)
Supplement: Unedited blot and gel images [file jci-134-163648-s098.pdf]

Figure S1 (related to Figure 1)

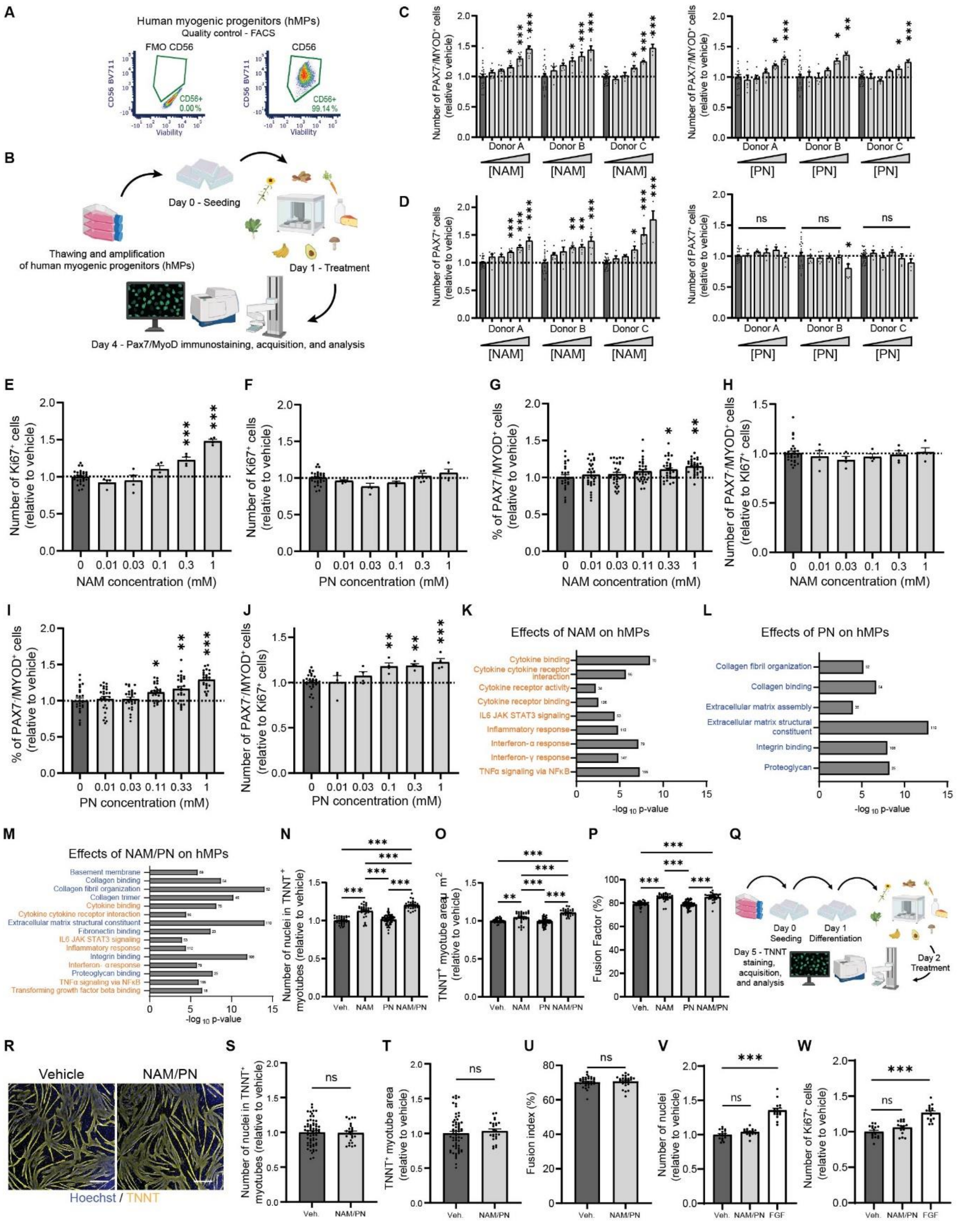

Fig. S1 (related to Figure 1): Effects of NAM and PN on hMP proliferation and differentiation. (**A**) Representative FACS plot of CD56 expression in hMPs. (**B**) Graphic overview of the high-content screening assay for PAX7$^+$ and MYOD$^+$ expression in hMPs. (**C,D**) Dose-response of NAM and PN on PAX7$^-$/MYOD$^+$ (C) and

$PAX7^+$ (D) hMPs. n ≥ 4 cell culture replicates from N = 3 donors. (**E**,**F**) Dose-response of NAM and PN (F) on $Ki67^+$ hMPs. n = 4 cell culture replicates from one donor. (**G-J**) Dose-response of NAM (G,H) and PN (I,J) on the percentage of $PAX7^-/MYOD^+$ and $MYOD^+$ hMPs. n ≥24 (G,I) or n = 4 for (H,J) cell culture replicates from N = 2 donors. (**K-M**) Downregulated pathways following NAM (K), PN (L), or NAM/PN (M) treatment of hMPs. A false discovery rate FDR of 10% was applied on all gene sets from KEGG and GO Terms (BP, Biological Process; CC, Cellular Component; and MF, Molecular Function) of MSigDB-enriched hMPs from 5 donors. (**N-P**) Quantification of the number of nuclei within myotubes (N), myotube area (O), and fusion index (P) following vehicle, NAM, PN or NAM/PN treatment during proliferation and differentiation. n ≥ 28 cell culture replicates from one donor. (**Q**) Graphic overview of the high-content assay for myotube differentiation of hMPs independent of proliferation. (**R-U**) Representative images (R) and quantification of the number of nuclei within myotubes (S), myotube area (T) and fusion index (U) after a 4 day-treatment with vehicle or NAM/PN in differentiating medium. Scale bar, 500 µm. n ≥ 24 cell culture replicates per condition from one donor. (**V,W**) Quantification of total number of nuclei (V) and Ki67+ cells (W) following NAM/PN treatment of non-myogenic human dermal fibroblasts. n≥16 cell culture replicates. FGF was used as a positive proliferation control. Data represented as means ± s.e.m. *** $P < 0.001$, ** $P < 0.01$, * $P < 0.05$ with one-way ANOVA followed by post hoc Dunnett's (C,D,E,F,G,H,I,J,V,W), Tukey's (N,O,P) multiple comparison test and two-tailed unpaired Student's t-test (S,T,U).

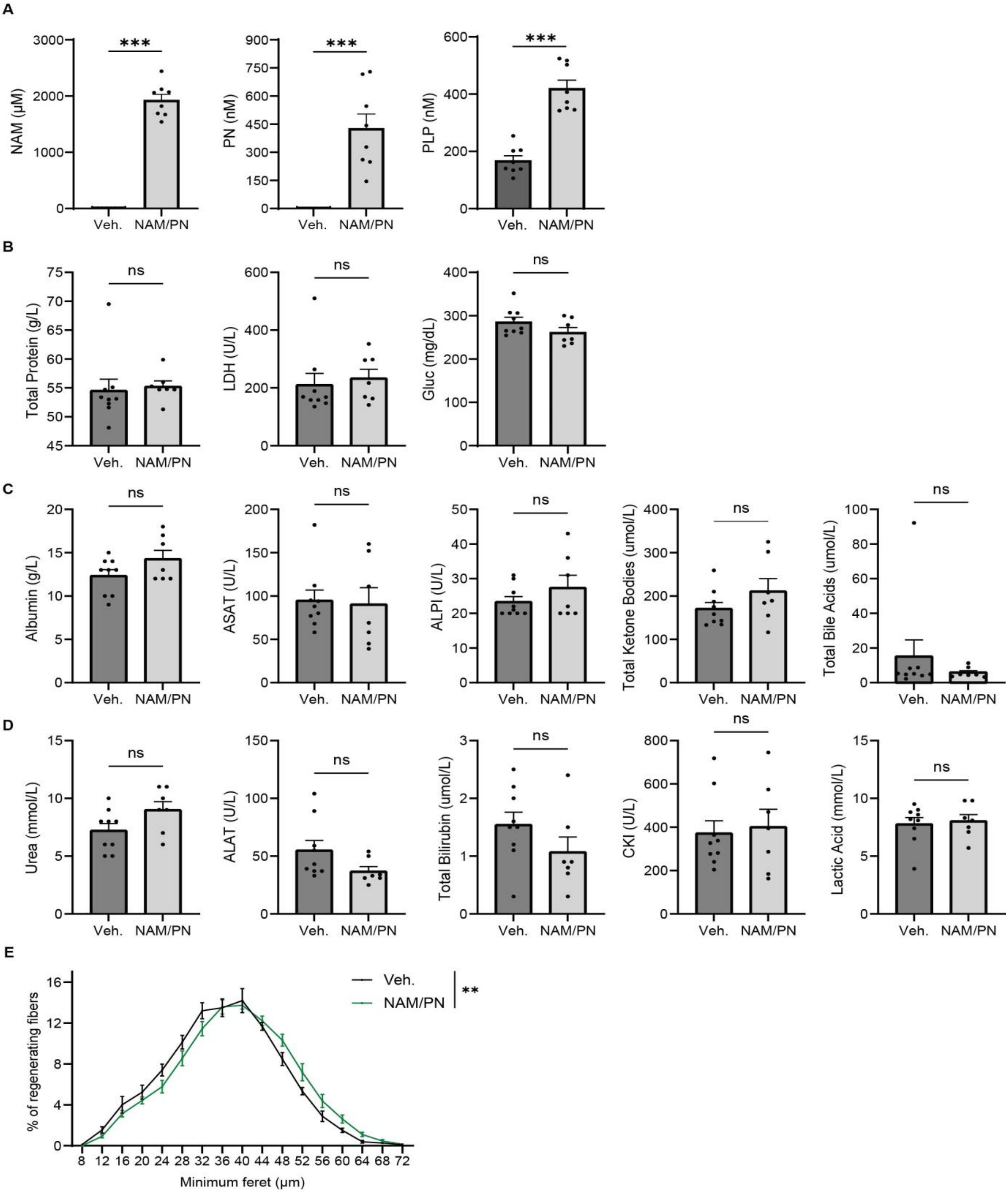

Fig. S2 (related to Figure 2): Plasma biomarkers following 12 days of daily oral supplementation of NAM/PN in young mice. (**A**) Quantification of NAM, PN, and Pyridoxal-5'-Phosphate (PLP), the active form of PN, in the plasma of vehicle- and NAM/PN-treated mice. (**B-D**) Quantification of standard plasma biochemistry profiles for safety assessment. (**E**) Distribution of regenerating myofiber size in injured *Tibialis Anterior* muscle of vehicle- and NAM/PN-treated mice 12 days post injury. N ≥ 5 mice per condition. Two-tailed unpaired Student's t-test. *** $P < 0.001$. Two-tailed unpaired Student's t-test (A, B, C, D) and Kolmogorov-Smirnov test to assess minimum fiber ferret distribution (E).

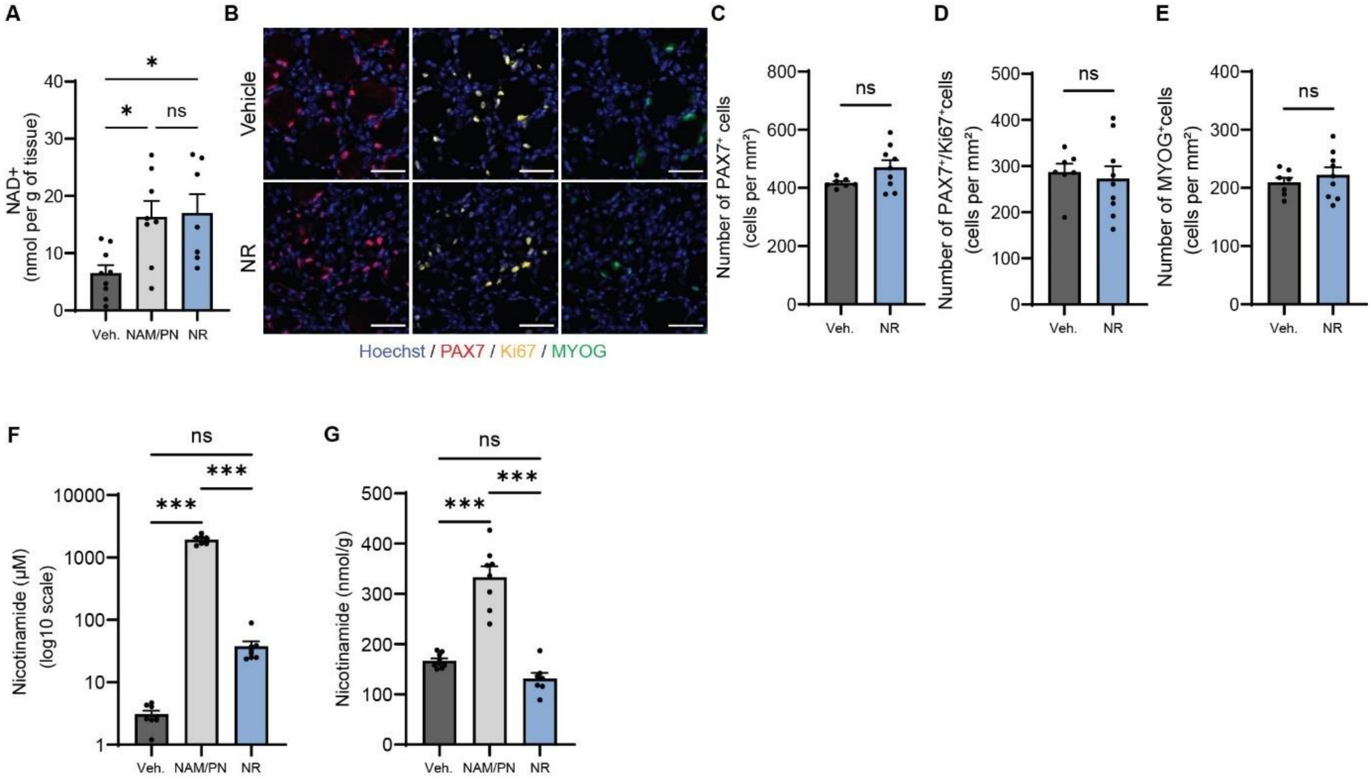

Fig. S3 (related to Figure 3): Effect of *in vivo* nicotinamide riboside supplementation on MuSCs during muscle regeneration. (**A**) Effect of NAM and NR on NAD^+ levels in gastocnemius muscle. (**B-E**) Representative images of *Tibialis Anterior* (TA) cross-sections in vehicle- and NR-treated mice (B) and quantification of $PAX7^+$ (C), $PAX7^+$/$Ki67^+$ (D), and $MYOGENIN^+$ (E) cells at 5 dpi. Scale bar, 50 µm. (**F-G**) LC-MS/MS quantification of NAM concentrations in plasma (F) and *Gastrocnemius* muscle (G) of NAM/PN-treated and NR-treated mice compared to vehicle-treated mice. Values for vehicle and NAM/PN correspond to the values presented in Fig. 2F,G and were reported here again to facilitate comparison with NR. Data from N ≥ 7 mice per condition represented as means ± s.e.m. All panels analyzed with PN co-treatment. *** $P < 0.001$, ** $P < 0.01$, * $P < 0.05$ with two-tailed unpaired Student's t-test (C,D,E) and one-way ANOVA with one-way ANOVA followed post hoc Tukey's multiple comparison test (A,F,G).

Figure S4 (related to Figure 4)

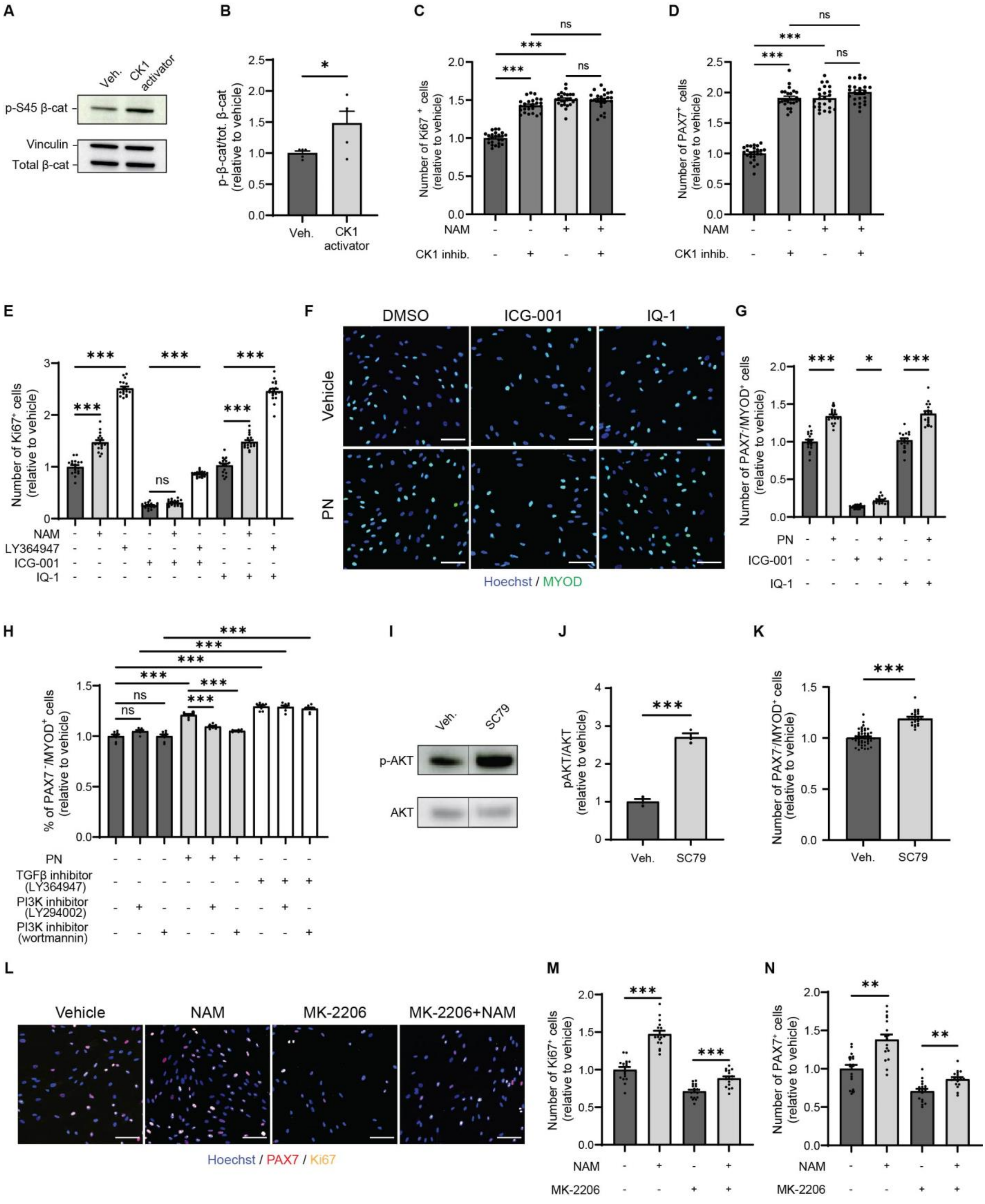

Fig. S4 (related to Figure 4): Effects of NAM and PN following inhibition of β-catenin and Akt signaling. (**A,B**) Representative immunoblot images (A) and quantification (B) of Ser45 phosphorylated β-catenin in hMPs treated with the CK1α activator pyrvinium. $n \geq 5$ cell culture replicates from one donor. **(C,D)** Quantification of the number of Ki67^{+} (C) and Pax7^{+} (D) by immunofluorescence of human myogenic progenitors (hMPs) after treatment with NAM or a of CK1α inhibitor. $n \geq 16$ cell culture replicates per condition from one donor. (**E**) Number of Ki67^{+} hMPs

after treatment with NAM or the positive control LY364947 in presence or absence of b-catenin inhibitors ICG-001 and IQ-1. n ≥ 17 cell culture replicates from one donor. **(F,G**) Representative immunofluorescence images (D) and quantification (E) of hMPs treated with vehicle, PN and/or the β-catenin nuclear inhibitors ICG-001 and IQ-1. Scale bar, 100 µm. n ≥ 17 cell culture replicates from one donor. **(H**) Percentage of $PAX7^-/MYOD^+$ hMPs after treatment with the PI3K inhibitors LY294002 and wortmannin for 72h in proliferation medium. Scale bar, 100 µm. n ≥ 7 cell culture replicates per condition from a single donor. (**I-K**) Representative immunoblot (I), quantification of pAkt to Akt ratio (H; n=3 cell culture replicates) and quantification of the number $MYOD^+$ cells (K; n ≥ 23 cell culture replicates per condition from one donor) in hMPs co-treated with the Akt activator SC79. (**L-N**) Representative immunofluorescence images (L) and quantification of $Ki67^+$ (M) and $PAX7^+$ (N) in hMPs treated with NAM and/or the allosteric Akt inhibitor MK-2206. n ≥ 17 cell culture replicates per condition from one donor. Data represented as means ± s.e.m. *** $P < 0.001$; ** $P < 0.01$; * $P < 0.05$ with two-tailed unpaired Student's t-test (B,J,K) and one-way ANOVA with Sidak's multiple comparisons adjustment (C,D,G,H,M,N).

Figure S5 (related to Figure 5)

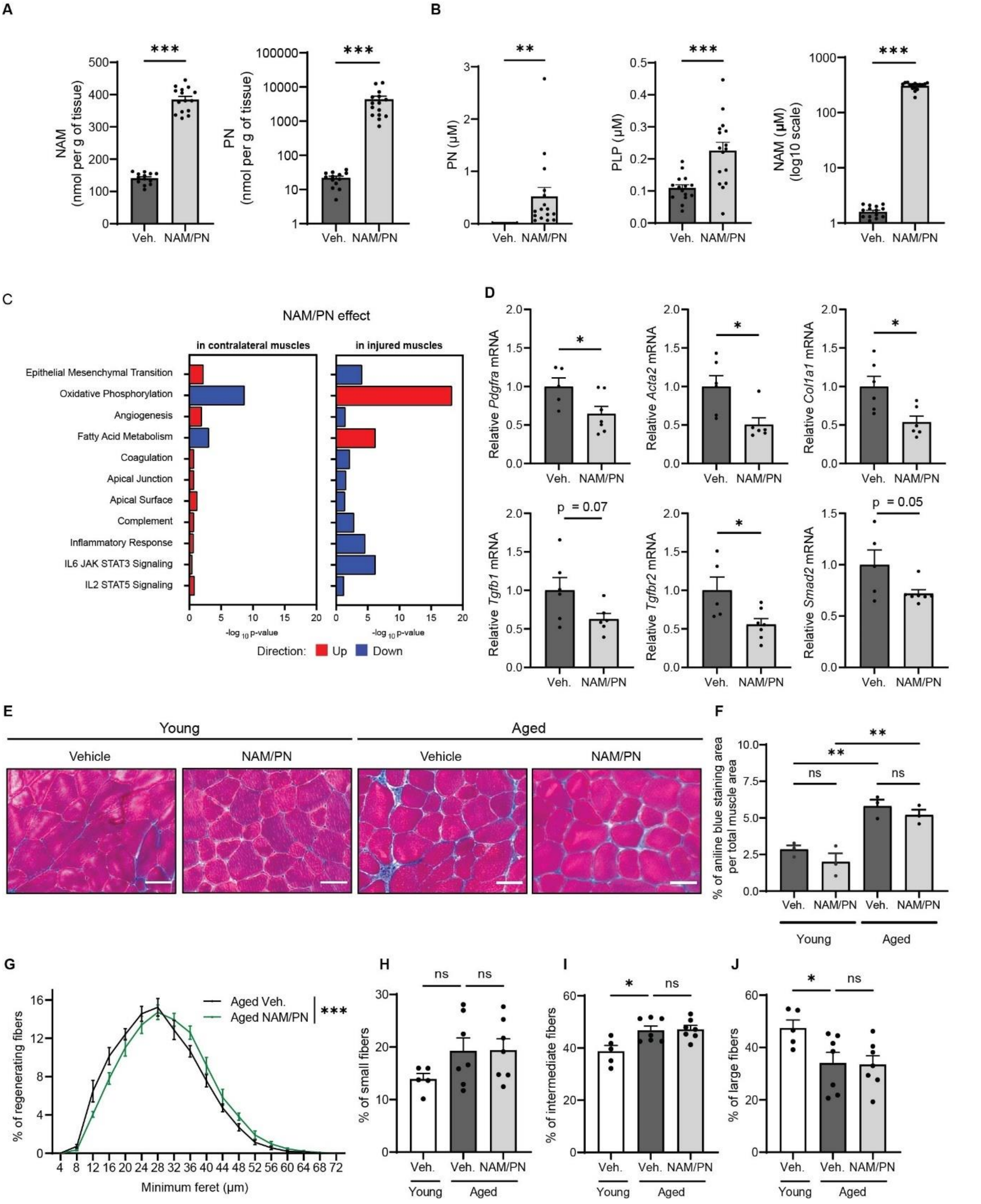

Fig. S5 (related to Figure 5): Effects of NAM/PN supplementation on plasma metabolites and muscle fibrosis in young and aged mice. (**A**) LC-MS/MS quantification of NAM and PN concentrations in uninjured *Gastrocnemius* muscles from aged vehicle (N = 13) and aged NAM/PN-treated mice (N = 15). (**B**) Quantification of NAM, PN, and PLP, in the plasma of aged vehicle (N = 15)- and aged NAM/PN-treated mice (N = 16). (**C**) Gene set enrichment analysis after RNAseq of contralateral and injured 5 dpi *Gastrocnemius* muscles of aged mice treated with NAM/PN.

To enable a direct comparison, data from Fig 5-I are also reported in this panel. A false discovery rate FDR of 10% was applied. (**D**) Quantification of fibrotic gene expression from regenerating *Gastrocnemius* muscles at 12 dpi from aged vehicle- ($N \geq 5$)- and NAM/PN-treated ($N \geq 5$) mice. (**E,F**) Representative images (E) and quantification (F) of Masson trichrome staining of cross-sections from uninjured muscles of young (N = 3) and aged (N = 3) mice. Quantification of the aniline blue fibrotic area was normalized to the total muscle area in cross-sections. (**G**) Distribution of regenerating myofiber size in injured *Tibialis Anterior* muscle of young and aged vehicle-treated (N = 7) and NAM/PN-treated mice (N = 6) at 12 dpi. (**H-J**) Quantification of minimum ferret size of small (≤ 33µm) (H), intermediate (>33µm and ≤ 44µm) (I), and large (>45µm) (J) myofibers of uninjured contralateral *Tibialis Anterior* muscle cross-sections from young (N = 5) and aged vehicle- (N = 7) and NAM/PN-treated (N = 7) mice. Data are represented as means ± s.e.m. *** $P < 0.001$, ** $P < 0.01$, * $P < 0.05$, using a two-tailed unpaired Student t-test (A, B, D), one-way ANOVA with post-hoc Dunnett test (H, I, J) , Tukey multiple comparison test (F) and Kolmogorov-Smirnov test (G). Scale bar, 50µm.

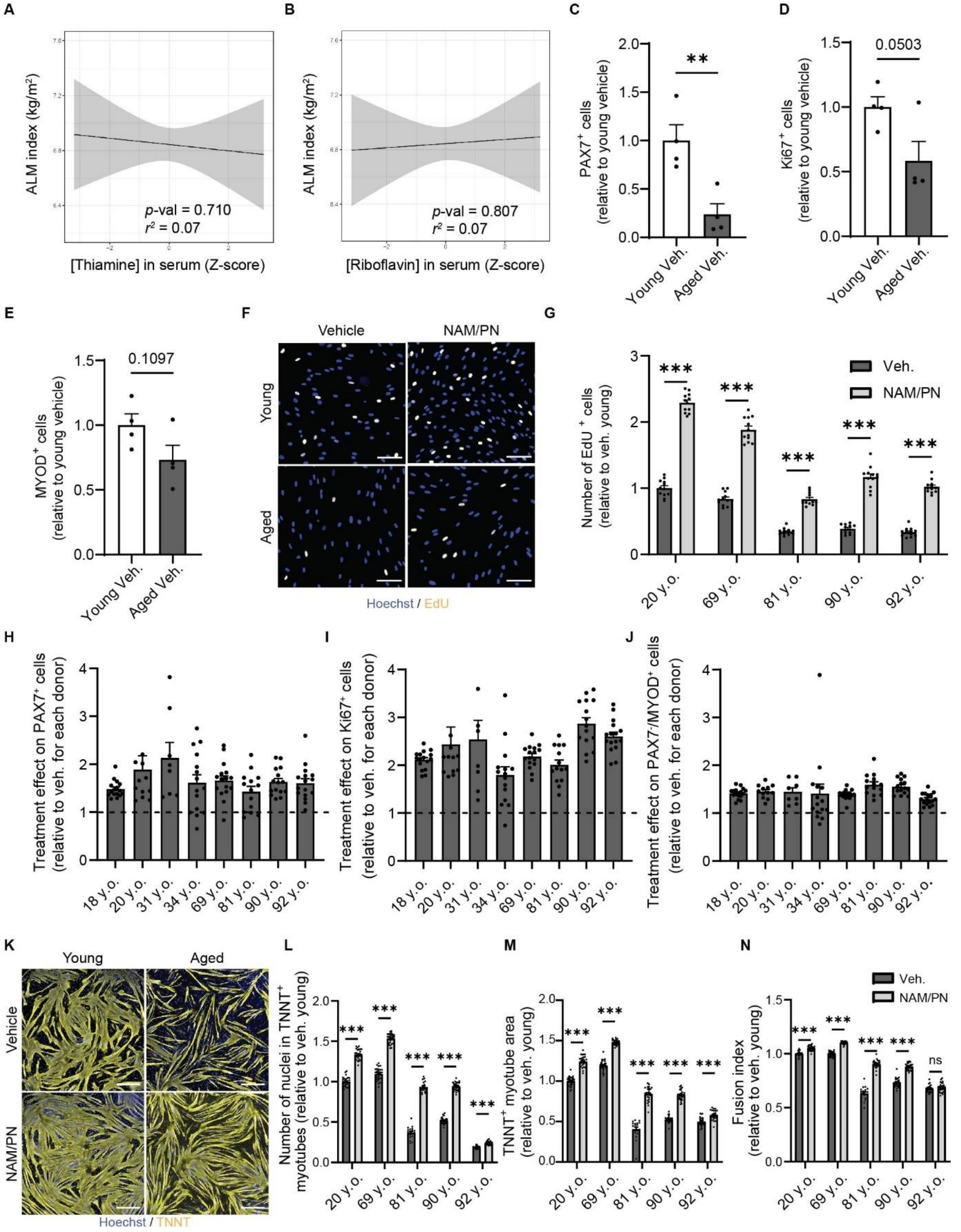

Fig. S6 (related to Figure 6): Clinical associations of unrelated B vitamins in older people and analysis of proliferation and differentiation of aged hMPs. (**A,B**) Appendicular lean mass index (ALMi) correlation with serum concentrations of Thiamine (A) and Riboflavin (B) using a simple linear regression model. Regression line

(black) and 95% confidence interval (gray). (**C-E**) Quantification of the number of PAX7$^+$ (C), Ki67$^+$ (D) and MYOD$^+$ (E) in vehicle-treated human myogenic progenitors (hMPs) from young (18-24y) and aged (69-92y) donors, n ≥ 12 cell culture replicates per condition from N = 8 donors following 72h of treatment with vehicle or NAM/PN. (**F-J**) Representative immunofluorescence pictures (F) and quantification (G) of EdU$^+$ hMPs from five donors aged from 20 to 92 years following treatment with vehicle or NAM/PN. n ≥ 12 cell culture replicates. (**H-J**) Fold change of PAX7 (H), Ki67 (I), and MYOD (J) positive cells following NAM/PN treatment from eight donors aged 18 to 92 years compared to their vehicle-treated condition (dotted black line). n ≥ 8 cell culture replicates per condition from N = 8 donors. Scale bar, 100 µm. (**K-N**) Representative images (K) and quantification of the number of nuclei within myotubes (L), myotube area (M), and fusion index (N) in hMPs from five donors aged from 20 to 92 years and treated with vehicle or NAM/PN for 72h in proliferation media and then switched to differentiation medium for 72 h. Scale bar, 500 µm. n ≥ 19 cell culture replicates per condition from N = 5 donors. Data represented as means ± s.e.m. *** $P < 0.001$; * $P < 0.05$; Data are mean ± SEM and were assessed with a two-tailed unpaired Student t-test (C,D,E) or Brown-Forsythe and Welch ANOVA tests with Dunnett T3 multiple-comparison (G,L,M,N).

List of supplementary tables available for download

Supplementary Table S1: List of hMP donors & corresponding figures

Supplementary Table S2: List of screening hits

Supplementary Table S3: List of gene set enrichment from hMP RNAseq

Supplementary Table S4: List of differentially expressed genes from hMP RNAseq

Supplementary Table S5: Association of clinical variables with serum metabolites, adjusted for age

Supplementary Table S6: Association of clinical variables with dietary intake and serum metabolites adjusted for dietary intake, all adjusted for age

SUPPLEMENTARY MATERIAL & METHODS

Primary human myogenic progenitors

For each experiment, a frozen stock of hMPs cells banked with less than 4 passages was thawed and expanded in proliferation medium (Skeletal Muscle Cell Growth Medium from AmsBio, #SKM-M) in a humidified incubator at 37°C in 5% CO2. hMPs were cultured for a maximum of 3 passages prior to cellular assays and were passaged upon reaching 50-60% confluence, approximately every 3 days.

hMP proliferation and commitment assays

An automated high throughput imaging phenotypic assay was developed to assess hMP proliferation and myogenic commitment in 384-well plates (Greiner, #781096). All liquid dispensing, compound treatment, and imaging steps were conducted on automated platforms optimized for this screen. hMPs were seeded at 800 cells per well in 384-well plates pre-coated with 10µg/ml human fibronectin (Corning, #356008) using a multi-drop automated dispenser (Thermo Fisher Scientific). 24 h after cell seeding, compounds were added using a fully automated customized liquid handling & cellular screening platform (Thermo Fisher Scientific). Cells were incubated for 72 h under humidified conditions at 37°C in 5 % CO2 and after three days, automated cell fixation and staining for nuclei, PAX7 (DHSB, 1/250) and MYOD (Novus Biologicals, #NBP1-54153, 1/500) was performed using an EL406 Washer/Dispenser (Biotek; see immunocytochemistry section for staining methods).

hMP differentiation assays

hMP cells were either treated with the NAM/PN combination during the proliferation and differentiation phases or only after differentiation was induced. In the first scenario, hMPs were seeded at 800 cells per well into 384-well plates pre-coated with 10µg/ml human fibronectin (Corning, #356008) and grown in Skeletal Muscle Cell Growth Medium (AmsBio, #SKM-M). 24 h after cell seeding, hMPs were treated with 1% DMSO (vehicle condition) (Sigma, #D4540) or the NAM/PN combination at a concentration of

1mM and 50 µM, respectively. Cells were cultured for 72 h in proliferation medium and differentiation was induced by switching to low-serum medium (DMEM (Thermo Fisher Scientific, #31331028) supplemented with 2% horse serum (Thermo Fisher Scientific, #26050088), 1% Penicillin-Streptomycin (Thermo Fisher Scientific, #15070063) supplemented either with vehicle or NAM/PN and cultured for another 4 days. In the second scenario, hMPs were seeded at 3,000 cells per well into 384-well plates pre-coated with 20µg/ml human fibronectin (Corning, #356008) and grown in Skeletal Muscle Cell Growth Medium (AmsBio, #SKM-M). 24 h after cell seeding, differentiation was induced as above with vehicle or NAM/PN treatment only during the differentiation phase.

hMPs immunocytochemistry and image acquisition

hMPs cells were fixed using 4% PFA and permeabilized in 0.1% Triton X-100 (Sigma, #T9284). Cells were blocked in 4% BSA (Sigma, #A8022) and stained with the following primary antibodies: anti-PAX7 (DHSB, 1/250), anti-MYOD (Novus Biological, #NBP1-54153, 1/500), anti-Ki67 (Thermo Fischer Scientific, #14-5698-82, 1/500), or anti-TNNT (Abcam, #ab45932, 1/2000) and then with appropriate secondary antibodies (Thermo Fisher Scientific, #A32733 and #A-21121; SouthernBiotech, #3065-32, 1/2000). Cell nuclei were counterstained with Hoechst 33342 (Sigma, #B2261, 1/20000). To assess hMP proliferation, 5µM EdU was added in the medium for 2h before fixation and EdU incorporation was revealed using the Click-iT assay (Thermo Fisher Scientific, #C10338) according to manufacturer's instruction. Briefly, cells were fixed during 15 minutes in 4% PFA, permeabilized during 20 minutes in PBTX 0.5%, stained with the Click-iT reaction mix and counterstained with Hoechst 33342 (Sigma, #B2261). Images were acquired using a high content imager (ImageXpress, Molecular Devices) equipped with a plate stacker (Spinnaker, Thermo Fisher Scientific) and quantifications were performed using the MetaXpress software (Molecular Devices). An exclusion of artifacts was performed based on morphological analysis and myotubes were detected based on the segmentation of TNNT staining. Multi-Wavelength Cell scoring was performed by identifying positive and negative populations based on a minimum intensity threshold for each marker using an automated image processing module.

In vivo experiments

All mice were randomized to different groups according to their weights. Muscle regeneration was induced on by intramuscular injection of 25µl and 50µl of 20µM cardiotoxin (CTX, Latoxan) into the *Tibialis anterior* (TA) and the *Gastrocnemius* (GC) muscles under a short anesthesia using 2% isoflurane. For analgesia, buprenorphine was administered at a dosage of 0.1mg/kg subcutaneously before the intramuscular injection followed either by a second dose 24h after or by administration through drinking water (at 9.4µg/ml) during 24h. Blood samples were collected in K2 EDTA tubes (BD Biosciences) by intracardiac puncture under a short terminal anesthesia using 2% isoflurane and plasma was prepared at 4°C and rapidly snap frozen in liquid nitrogen. Mice were sacrificed by CO2 exposure 5- or 12-days post-injury and contralateral and regenerating muscles were quickly collected and processed. 2/3 of the TA was mounted on 6% tragacanth gum (Sigma, #G1128) and frozen in isopentane (Sigma, #M32631) cooled with liquid nitrogen for histological analysis, and the rest of the TA and GC muscles were snap-frozen in liquid nitrogen for molecular analyses. For *in vivo* contraction assays to induce eccentric contraction-induced regeneration and measure muscle strength longitudinally, mice were anesthetized in an induction chamber using 4% isoflurane. The right leg was shaved before an electrode cream was applied at the knee and heel regions to optimize electrical stimulation. Each anesthetized mouse was placed in a supine position in a non-invasive specific device (NIMPHEA_Research, All Biomedical SAS, Grenoble, France). Anesthesia was maintained by gas inhalation through as facemask continuously supplied with air flow 0.7 L/min and 2-2.5% isoflurane. The right leg was stabilized and placed on two fixed electrodes: one under the knee and one on Achille's tendon to activate the plantar flexor muscles. The right foot was firmly immobilized through a rigid slipper on a pedal connected to a torque sensor allowing for plantar flexor torque measurement. The foot was set at an angle of 20° of plantarflexion. The right knee was also firmly maintained using a rigid fixation in order to optimize isometric torque recordings and muscle damage.

Single transcutaneous stimulations on the plantar flexors were delivered using a constant current stimulator (Digitimer DS7AH, Hertfordshire, UK; maximal voltage: 400 V; 0.2 ms duration, monophasic rectangular pulses). The individual maximal current intensity was determined by progressively increasing the current intensity until there was no further peak twitch torque increase (maximal current intensity ranged from 120

to 150 mA). Three single twitches were delivered at this intensity with a 8-sec resting period in between. Then, mice were submitted to 30 maximal electrically-evoked lengthening contractions every 10 sec and evoked at 100 Hz (175-ms duration, stretching was initiated 150 ms after the onset of stimulation) from 20° to -10° at an angular velocity of 800°/s which was controlled by a servomotor. A plastic tie was wrapped around the rigid slipper to avoid any foot movement during the protocol. Mice were then returned to cages. Isometric peak twitch torque measurements were repeated at days 1, 7, and 14 and *gastrocnemius* muscles were collected for histology.

Human nutritional epidemiology study (Bushehr Elderly Health)

Dietary intake assessment was performed by a 24-hour dietary recall of all food and beverages consumed, captured performed by expert nutritionists (*1*). Standard reference tables were used to convert household portions to grams and quantified using the nutritionist IV package, modified for Iranian foods to obtain daily energy, nutrient values and servings of foods consumed. For mixed dishes, food groups and nutrients were calculated according to their ingredients.

Histology of muscle sections and image analyses

Frozen muscles were cryo-sectioned into 10μm thick muscle cross-sections using a cryostat (Leica Biosystems) and stored at -80°C. For PAX7, MYOGENIN, and Ki67 staining, slides were fixed with 4% PFA (EMS, #157-4-100), and permeabilized in cold methanol (VWR, #1.06035.2500). Antigen retrieval was performed with two successive incubations of hot 0.01 M pH 6 citric acid during 5 min, and sections were further blocked in 4% BSA for 2 h, followed by 30 min blocking with an anti-mouse FAB (Jackson #115-007-003, 1/100). We next incubated the slides with primary antibodies anti-PAX7 (DHSB, purified, 2.5 µg/ml), anti- MYOGENIN (Abcam, #ab124800, 1/500), and anti-Ki67 (Thermo Fischer Scientific, #14-5698-82, 1/500). PAX7 signal was further amplified using a goat-anti mouse IgG1-biotin (Jackson ImmunoResearch #115-065-205, 1/1000) followed by conjugation with Streptavidin Alexa555 (Life Technologies, #S-21381, 1/2000), and other antibodies were detected with their specific secondary

antibodies (Thermo Fisher Scientific, #A32731; SouthernBiotech, #3065-31), while nuclei were detected with Hoechst 33342 (Sigma, #B2261). Slides were then mounted using Dako fluorescent mounting medium (Agilent, #S302380-2) and imaged with an Olympus VS120 slide scanner. Images were analyzed using the VS-ASW FL software measurement tool. The number of PAX7, MYOGENIN, and Ki67 positive cells in the injured region was determined by manually counting several random areas on entire muscle sections with researchers blinded to the experimental groups. A minimum of 300 PAX7^{+} cells were quantified from at least 0.5 mm^2 (> 75%) of injured tissue for each mouse. For laminin immunostaining, cryosections were blocked for 45 min at room temperature in 4% BSA. Cryosections were stained for 3 h at room temperature using anti-laminin antibody (Sigma, #L9393, 1/1000). Sections were then incubated for 1 h with secondary antibodies (Thermo Fisher Scientific, #A32731) and Hoechst (Sigma, #B226). The size of myofibers was calculated across the entire sections on all fibers with centralized nuclei using an automated image processing algorithm developed internally using the QuPath software and the Fiji open-CSAM tool (*2*). For Masson Trichrome staining, frozen sections were dried and fixed 1 h with 4% PFA and then incubated overnight in Bouin solution at room temperature. Slides were rinsed under tap water for 30 min followed by dH_2O (30 sec) and stained in Weigert's hematoxylin solution for 5 min. Slides were successively dipped in water (1 min), 1% HCl diluted in 100% Ethanol (3 sec), in water again (10 min) and in 1% acetic acid (1 min). Slides were then stained in Biebrich scarlet-acid fuchsin solution (5 min). Slides were successively incubated in 1% acetic acid (2 min), 2.5% phosphomolybdic-2.5% phosphotungstic acid solution (10 min) and in 1% acetic acid (2 min). Slides were stained in 2.5% Aniline blue (5 min) and differentiated in 1% acetic acid (2x 1 min). Finally, slides were dehydrated in Ethanol 100% (2x 1 min) and cleared in Xylene (2x 1 min) before being mounted with Pertex mounting medium. Stained sections were acquired using an Olympus VS120 slide scanner and analyzed with Fiji. The fibrotic area was evaluated by quantifying the Aniline blue-positive area in uninjured muscle or in regenerating areas identified by the presence of fibers with centralized nuclei.

FACS-isolated mouse MuSCs

Freshly-sorted mouse MuSCs were isolated as previously described (*3*). Hindlimb muscles from uninjured young mice were rapidly collected, minced, and digested with Dispase II (2.5 U/ml) (Sigma, #04942078001), Collagenase B (0.2%) (Sigma, #1088807001) and MgCl2 (5 mM) at 37°C. The preparation was then filtered sequentially through 100 micron and 30 micron filters and cells were incubated at 4°C for 30 min with antibodies against CD45 (Invitrogen, #MCD4528, 1/25), CD31 (Invitrogen, #RM5228, 1/25), CD11b (Invitrogen, #RM2828, 1/25), CD34 (BD Biosciences, #560238, 1/60), Ly-6A/E (BD Biosciences, #561021, 1/150) and α7-integrin (R&D Systems, #FAB3518N, 1/30). MuSCs identified as $CD31^-$/$CD11b^-$/$CD45^-$/$Sca1^-$/$CD34^+$/Integrin $\alpha7^+$ were isolated with a Beckman Coulter Astrios Cell sorter.

Mouse MuSC proliferation assays and immunocytochemistry

FACS-isolated MuSCs from young (3 month-old) or aged (23-24 month-old) mice were directly plated onto gelatin-coated 384-well plates and grown over 4 days in DMEM supplemented with 20% heat-inactivated FBS (Thermo Fisher Scientific, #16140063), 10% horse serum (Thermo Fisher Scientific, #26050088), 2.5ng/ml bFGF (Thermo Fisher Scientific, #PMG0035), 1% Penicillin-Streptomycin (Thermo Fisher Scientific, #15070063) and 1% L+-Glutamine (Thermo Fisher Scientific, #25030149) and treated with the NAM/PN combination and with 0.5% DMSO as the vehicle condition. To assess MuSC proliferation, 5µM EdU was added in the medium the fourth day after sorting for 2h. EdU incorporation was revealed using the Click-iT assay (Thermo Fisher Scientific, #C10338) according to manufacturer's instruction. Briefly, cells were fixed during 15 minutes in 4% PFA (Electron Microscopy Sciences, #157-4-100), permeabilized during 20 minutes in Phosphate Buffered Saline (PBS, Thermo Fisher Scientific, #10010023) containing 0.5% triton (Sigma, #T9284), stained with the Click-iT reaction mix and counterstained with Hoechst 33342 (Sigma, #B2261). To access MuSC fate, PAX7 and MYOD immunostaining were performed by blocking for 1h in 4% BSA (Sigma, #A8022) and staining with anti-PAX7 (DHSB, 1/250), anti-MYOD (Labforce, # LS-C143580-100, 1/500), and then with appropriate secondary antibodies (Thermo Fisher Scientific, #A-21121 and #A32733, 1/2000). Images were acquired

using the ImageXpress (Molecular Devices) platform and quantifications were performed using the MetaXpress software (Molecular Devices). An exclusion of artifacts was performed based on morphological analysis and Multi-Wavelength Cell scoring was performed by identifying positive and negative populations based on a minimum intensity threshold for each marker.

Fibroblast proliferation assays

Similarly to hMP proliferation, human primary fibroblasts (HDF, Cell Applications, #106-05a) were seeded at 800 cells per well in 384-well plates using a multi-drop automated dispenser. Cells were cultured in growth medium DMEM (Thermo Fisher Scientific, #11966025) supplemented with 5mM glucose (Thermo Fisher Scientific, #A2494001), 10% fetal bovine serum (Thermo Fisher Scientific, #26050088), 1% Penicillin-Streptomycin (Thermo Fisher Scientific, #15070063). 24 h after cell seeding, compounds were added using a fully automated customized liquid handling & cellular screening platform. Cells were in growth medium containing DMSO, NAM/PN and 150ng/ml FGF (Sigma, #F8424) as positive control and incubated for 72 h under humidified conditions at 37°C in 5 % CO2. Cell fixation and staining for nuclei, Ki67 (Thermo Fischer Scientific, #14-5698-82, 1/500) was performed using an EL406 Washer/Dispenser. Automated imaging was performed in the fixed 384-well plates using a high content imager and image analysis was performed with the MetaXpress software (Molecular Devices) using the Multi-Wavelength Cell scoring automated module.

Metabolomics analyses

20-30mg of frozen muscle from mice was analyzed using LC/MS (MS Omics, Denmark) on a UPLC system (Vanquish, Thermo Fisher Scientific) coupled to a high-resolution quadrupole-orbitrap mass spectrometer (Q Exactive™ HF Hybrid Quadrupole-Orbitrap, Thermo Fisher Scientific). The analysis was carried out in both negative and positive ionization mode using a heating electrospray ionization interface. QC samples were injected at a regular interval to ensure signal stability throughout the sequence and finally a QC sample was analyzed in MS/MS mode for identification of compounds. Metabolites were quantified using an eight-

point calibration series (0.005 µM – 50 µM). Data were processed using Compound Discoverer 3.1 (Thermo Fisher Scientific) and TraceFinder 4.1 (Thermo Fisher Scientific). The following metabolites were quantified: NAD, nicotinamide (NAM), pyridoxine (PN), and pyridoxal-5'-phosphate (PLP). Mouse plasma or human serum from the Bushehr Elderly Health (BEH) rapidly processed and stored at -80°C was used for circulating metabolomic analysis. Concentrations of nicotinamide (NAM), pyridoxal 5'-phosphate (PLP, bioactive from of PN) and pyridoxine (PN) were measured using LC-MS/MS by BEVITAL (Bergen, Norway; www.bevital.no). Measurements were performed by mixing samples with labelled internal standards, separation on a C8 liquid chromatography column by a gradient-type mobile phase and detecting analytes using electrospray ionization tandem mass spectrometry as described in (*4*).

Plasma biochemistry

Plasma biochemistry was measured on mouse plasma diluted 1:1 using Dimension®Xpand Plus (Siemens Healthcare Diagnostics AG). The biochemical tests were performed according to the manufacturer's instruction for each parameter: Albumin (Siemens Healthcare, #DF13), Alcaline Phosphatase (Siemens Healthcare, #DF15A), Amylase (Siemens Healthcare, #DF17A), Bilirubin total (Siemens Healthcare, #DF167), Creatine Kinase (Siemens Healthcare, #DF38), Glucose (Siemens Healthcare, #DF40), Lactic acid (Siemens Healthcare, #DF16), Transaminase ASAT (Siemens Healthcare, #DF41A), transaminase ALT (Siemens Healthcare, #DF143), Urea Nitrogen (Siemens Healthcare, #DF21), Total protein (Siemens Healthcare, #DF73), Total Bile acid (Diazyme Laboratories, #DZ042A-K), Total Ketone Bodies (FUJIFILM Wako Diagnostics, Autokit TKB #k415-73301).

Quantitative RT-PCR on muscles

RNA was isolated from snap frozen GC muscle. 10-15mg were transferred to lysing matrix tubes (MP Biomedicals) containing QIAzol (Qiagen, #79306), and homogenized with a FastPrep® (MP Biomedicals). Chloroform (Sigma) was added to the mixture, centrifuged for 15 min at 4°C to allow phase separation, and the clear phase containing RNA was transferred in new tubes and processes with automated RNA isolation using the RNeasy Mini Kit (Qiagen) program on a QIAcube (Qiagen). cDNA was reverse-

transcribed from 1.5 µg of RNA using the High-Capacity cDNA Reverse Transcription Kit (Thermo Fisher Scientific) following manufacturer's instructions, and cDNAs were diluted 1:10 in dH_2O to perform quantitative RT-PCR on a LightCycler® 480 (Roche) using SYBR Green PCR Master Mix (Roche). The pre-incubation phase was performed at 95°C for 7 min and cDNAs were then amplified over 45 cycles at 95°C for 15 s and 63°C for 1 min. To quantify mRNA expression, $2^{-\Delta\Delta Ct}$ calculation was used to express results relative to *Atp5b*, *Eif2a*, and *Psmb4* expression as these reference genes were previously selected based on their stability across time points of regeneration (*16*). The following primer sequences were used: *Atp5b*, forward: 5'-ACCTCG-GTGCAGGCTATCTA-3', reverse: 5'-AATAGCCCGGGACAACACAG-3'; *Eif2a*, forward: 5'-CACGGTGCTTCCCAGAGAAT-3', reverse: 5'-TGCAGT-AGTCCCTTGTTAGCG-3'; *Psmb4*, forward: 5'-GCGAGTCAACGACAGCACTA-3', reverse: 5'-TCATCAATCACC-ATCTGGCCG-3'; *Pdgfra*, forward: 5'-AGTGGC-TACATCATCCCCCT-3', reverse: 5'-CCGAAGTCTGTGAGCTGTGT-3'; *Col1a1*: 5'-CGATGGATTCCCGTTCGAGT-3', reverse: 5'-GAGGCCTCGGTGGACATTAG-3'; *Acta2* (αSMA), forward: 5'-CAGTCGCTGTCAGGAACCC-3', reverse: 5'-GGAGCATCATCA-CCAGCGAA-3'; *Tgfb1,* forward: 5'-GGAGACGGAATACAGGGCTT-3', reverse: 5'-GGCTGATCCCGTTGATTTCC-3'; Tgfbr2, forward: 5'-ACGTGGAGTCGTTCAAGCAG-3', reverse: 5'-AATCCTTCACTT-CTCCCACAGC-3'; *Smad2*, forward: 5'-CGTCCA-CTCTTGCCATTCAC-3', reverse: 5'-GTCCATTCTGCTCTCCACCA-3'.

Protein extraction and immunoblotting

For Western Blots, hMPs were plated on fibronectin-coated 6-well plates at a density of 100,000 cells per well and grown for 48 h in Skeletal Muscle Cell Growth Medium (AmsBio, #SKM-M). Cells were treated with 1 mM of PN, 1 µM of MK-2206 (Selleckchem, #S1078), or both in medium containing 1% DMSO. Proteins were extracted in RIPA lysis and extraction buffer (Thermo Fisher Scientific, #89901) supplemented with protease (Sigma, #S8820) and phosphatase inhibitor cocktail (Sigma, #4906845001). Protein concentration was determined using a BCA assay (Thermo Fischer Scientific, #23227). Samples were first diluted to 1.5mg/mL and boiled 5 min in NuPAGE™ LDS Sample Buffer (4X) (Invitrogen, #NP0007), run on 4–12% Bis-Tris Protein gels (Thermo Fischer Scientific, #BN1003), and transferred

using the semi dry system from Life Technologies. Membranes were incubated overnight at 4°C with primary antibodies anti-phospho-Akt (Ser473) (Cell Signaling, #4060, 1/1000), anti-Akt (Cell Signaling, #9272, 1/1000), anti-Phospho-β-Catenin (Ser675) (Cell Signaling, #4176, 1/1000), anti-β-Catenin (Cell Signaling, #8480, 1/1000) and anti-Vinculin (Cell Signaling, #13901, 1/5000). Membranes were then washed and incubated for 1 h with horseradish peroxidase–conjugated donkey anti-rabbit (Jackson ImmunoResearch, #711-035-152, 1/5000). Proteins were visualized with chemiluminescent western blotting substrate (Thermo Fisher Scientific, #32132) using Amersham Hyperfilm™ films or e-BLOT Touch Imager (e-BLOT Life Science). Densitometry analysis was performed using Fiji. Protein levels in each lane were normalized to the levels of vinculin as a loading control. Capillary-based Simple Western was performed on purified nuclear fractions from hMPs. Cells were treated for 48 h with 1 mM NAM and 200 ng/ml Wnt3a (R&D Systems, 5036-WN-010/CF) in medium containing 1% DMSO. Nuclear extraction was performed using the NE-PER Nuclear Cytoplasmic Extraction Reagent kit (Thermo Fisher Scientific, #78833) according to the manufacturer's instructions. Purified nuclear extracts were quantified using a BCA Protein Assay (Thermo Fischer Scientific, #23227). Nuclear extract concentrations were next normalized and diluted in Simple Western Dilution Buffer and mixed with Fluorescent Standard/4X Master Mix (ProteinSimple, #DM-001). All reagents from Protein Simple, including blocking reagent, primary antibodies, HRP-conjugated secondary antibodies, Luminol and peroxide substrate, separation and stacking matrices were dispensed into the 384-well plate according to the manufacturer's instructions. Separation electrophoresis and immunodetection then occurred automatically. Primary antibodies directed to β-catenin (Cell Signaling, #8480, 1/100), non-phosphorylated (active) β-catenin (Cell Signaling, #8814, 1/10), acetyl-Lys49-β-catenin (Cell Signaling, #9030, 1/100) and lamin A/C (Cell Signaling, #4777, 1/50) were used. Data were analyzed using the Simple Western Compass software. Protein expression was determined by quantifying the area under the curve (AUC) after a normalization to laminA/C used as a loading control.

NAD^+ Content

NAD^+ from hMPs was quantified using the NAD/NADH Assay Kit (Sigma, #MAK037) according to the manufacturer's instructions. Briefly, hMPs were plated on fibronectin-coated 6-well plates at a density of

100,000 cells per well and grown for 72 h in Skeletal Muscle Cell Growth Medium (AmsBio, #SKM-M) containing 1% DMSO, 1mM of NAM, 1mM NMN (β-Nicotinamide mononucleotide, Sigma, #N3501), 1mM NR (Nicotinamide Riboside Chloride, ChromaDex, #00014332), 100µM of FK-866 (Sigma, #F8557), 100µM of NAM (NAM^{Low}), or 1mM of NAM (NAM^{High}). hMPs were then lysed in NAD/NADH Extraction Buffer, and after centrifugation, the NAD/NADH-containing supernatant was transferred into a new tube and protein concentration was determined using a BCA assay (Thermo Fischer Scientific, #23227). Samples were diluted in NAD/NADH Extraction Buffer and NAD Cycling Mix was used to convert NAD to NADH. Finally, NADH Developer was added to each sample and the absorbance at 450 nm was measured on a Flexstation (Molecular Devices). NAD content was normalized to the total quantity of proteins.

CK1α activity measurement

A biochemical assay using purified recombinant CK1α was performed following instructions from the supplier (Promega, #V4484). Briefly, the reaction was prepared with 5µL total volume containing 10ng CK1α1, 2.5µg Casein, 25µM ATP, CK1 inhibitor TAK-715 (TOCRIS, #4254) or a range of NAM doses in reaction buffer and incubated at room temperature for 1h. Then, 5µL ADP-GloTM reagent was added for 40 min at room temperature. Next, 10µL of kinase detection reagent was added and incubated at room temperature for 30min. Finally, luminescence was recorded on a Synergy Neo plate reader (Biotek). The percentage of CK1α1 activity was calculated after removing the background of the reaction without CK1α1 from all measures.

Luciferase assay measurement

Primary MuSCs were isolated from males using magnetic-activated cell sorting method (MACS) following the manufacturer's instructions. Magnetic cell separation was performed on LD columns (Miltenyi). Primary MuSC were grown in 48-wells plates and cells in each well were co-transfected with 450ng of TopFlash or FopFlash reporter plasmid and 45ng of Renilla luciferase reporter as control. Plasmids were

transfected with Lipofectamine Stem reagent (Thermo Fisher Scientific, # STEM00001) following provider's instruction. Luciferase activity was measured using the Dual-Luciferase reporter assay system (Promega, # E1910). The TCF transcriptional activity is shown as a ratio of TopFlash to FopFlash luciferase-mediated signals.

RNA-Sequencing of hMPs, MuSC and gastrocnemius muscles

RNA was extracted from hMPs (donors A, C, D, I, J), GC muscles, and primary MuSCs isolated from young and aged mouse hindlimbs using the Agencourt RNAdvance Tissue Kit (Beckman Coulter#A32646). For hMPs (Figure 1F-I), libraries were constructed from cDNA amplified from 50ng following the user guide QuantSeq 3' mRNA-Seq (Lexogen 15.384). For GC muscles (Figures 5I and S5C), aged MuSCs treated ex vivo with vehicle or NAM/PN (Figure 5H, treatment effect), libraries were constructed from cDNA generated and amplified from 250 ng and 30 ng of RNA respectively, following the user guide QuantSeq 3' mRNA-Seq (Lexogen 15.384). For freshly isolated MuSCs from young *vs* aged mice (Figure 5H, age effect) sequencing libraries were prepared using the Ovation SoLo from NuGen from 2ng of RNA. All libraries were constructed following the recommendations from the respective kits without any modification. Libraries were quantified with Quant it Picogreen (Invitrogen, #10545213). The library sizes were controlled with the High Sensitivity NGS Fragment Analysis kit on a Fragment Analyzer (Agilent DNF-474-0500) and the DNA High Sensitivity Reagent kit on a LabChip GX (Perkin Elmer). The sequencing was performed on HiSeq 2500 or on NextSeq 2000. Primary data QC was performed during the sequencing run to ensure the optimal flow cell loading (cluster density) and check the quality metrics of the sequencing run.

RNA-Sequencing statistics

Sequencing data were demultiplexed with Bcl2FastQ and transformed into fastq files using casava v1.8.2. The fastq files were then aligned against the reference genomes GRCh38 for human samples and GRCm38 for mouse samples using RNAstar v2.5.3a (*5*). The counts per gene from the bam files were generated using htseq_count v2.16.2 (*6*).

For the human samples, after removing low expressed genes using the edgeR (*7*) function filterByExpr with the default parameters and specifying the sample treatments, counts per gene data were normalized by the trimmed mean of M-values (TMM) method as implemented in function calcNormFactors in edgeR. The voomWithQualityWeights function in LIMMA was applied to model the mean-variance relationship and estimate the sample-specific quality weights (*8*). To account for the repetitive nature of the dataset, correlations between repeated measurements from the same donor were estimated using the duplicateCorrelation method (*9*) that was estimated twice. Differentially expressed genes were identified using linear modelling and moderating the t-statistics by empirical Bayes shrinkage. To compare the number of DE genes found for 3 different contrasts (NAM-CTRL, PN-CTRL, and NAM/PN-CTRL), as reported in the Venn diagram, we applied the "global" method (*10*) as implemented in function decideTests. The main advantage of the "global" method is that a unique raw p-value cutoff is applied across all contrasts, this was set at 5% false discovery rate (FDR). Pathway enrichment analysis was performed using CAMERA (*9*), a competitive gene set test querying whether a set of genes annotated in the Molecular Signatures Database (MSigDB) is enriched in differentially expressed genes (*10*). KEGG pathways from MSigDB, C2 (curated gene sets) collection, and gene ontology biological processes from MSigDB, C5 (GO BP gene sets) were accessed through the library msigdbr 7.5.1 and used to perform pathway analyses.

For the mouse muscles, after removing low expressed genes using the edgeR function filterByExpr with default parameters and specifying the mouse groups, counts per gene data were normalized by the trimmed mean of M-values (TMM) method as implemented in function calcNormFactors in edgeR. Differentially expressed genes were defined by fitting a quasi-likelihood negative binomial generalized log-linear model to count data using glmQLFTest function in edgeR. Mouse-ortholog hallmark gene sets from MSigDB were accessed using the library msigdbr 7.5.1 and pathway enrichment analysis was performed using CAMERA.

SUPPLEMENTARY REFERENCES

1. G. Shafiee, A. Ostovar, R. Heshmat, H. Darabi, F. Sharifi, A. Raeisi, N. Mehrdad, Z. Shadman, F. Razi, M. R. Amini, S. M. Arzaghi, H. A. Meybodi, A. Soltani, I. Nabipour, B. Larijani, Bushehr Elderly Health (BEH) programme: study protocol and design of musculoskeletal system and cognitive function (stage II). *BMJ Open* **7**, e013606 (2017).

2. T. Desgeorges, S. Liot, S. Lyon, J. Bouvière, A. Kemmel, A. Trignol, D. Rousseau, B. Chapuis, J. Gondin, R. Mounier, B. Chazaud, G. Juban, Open-CSAM, a new tool for semi-automated analysis of myofiber cross-sectional area in regenerating adult skeletal muscle. *Skelet Muscle* **9**, 2 (2019).

3. L. Lukjanenko, M. J. Jung, N. Hegde, C. Perruisseau-Carrier, E. Migliavacca, M. Rozo, S. Karaz, G. Jacot, M. Schmidt, L. Li, S. Metairon, F. Raymond, U. Lee, F. Sizzano, D. H. Wilson, N. A. Dumont, A. Palini, R. Fässler, P. Steiner, P. Descombes, M. A. Rudnicki, C. M. Fan, J. von Maltzahn, J. N. Feige, C. F. Bentzinger, Loss of fibronectin from the aged stem cell niche affects the regenerative capacity of skeletal muscle in mice. *Nat Med* **22**, 897–905 (2016).

4. Ø. Midttun, S. Hustad, P. M. Ueland, Quantitative profiling of biomarkers related to B-vitamin status, tryptophan metabolism and inflammation in human plasma by liquid chromatography/tandem mass spectrometry. *Rapid Commun Mass Spectrom* **23**, 1371–9 (2009).

5. A. Dobin, C. A. Davis, F. Schlesinger, J. Drenkow, C. Zaleski, S. Jha, P. Batut, M. Chaisson, T. R. Gingeras, STAR: ultrafast universal RNA-seq aligner. *Bioinformatics* **29**, 15–21 (2013).

6. S. Anders, P. T. Pyl, W. Huber, HTSeq--a Python framework to work with high-throughput sequencing data. *Bioinformatics* **31**, 166–9 (2015).

7. M. D. Robinson, D. J. McCarthy, G. K. Smyth, edgeR: a Bioconductor package for differential expression analysis of digital gene expression data. *Bioinformatics* **26**, 139–40 (2010).

8. C. W. Law, Y. Chen, W. Shi, G. K. Smyth, voom: Precision weights unlock linear model analysis tools for RNA-seq read counts. *Genome Biol* **15**, R29 (2014).

9. G. K. Smyth, J. Michaud, H. S. Scott, Use of within-array replicate spots for assessing differential expression in microarray experiments. *Bioinformatics* **21**, 2067–2075 (2005).

10. G. Smyth, M. Ritchie, N. Thorne, J. Wettenhall, W. Shi, Linear Models for Microarray Data User’s Guide. (2010).

UNEDITED WESTERN BLOTS

Representative unedited western blots from Fig.4K and Fig.S4I

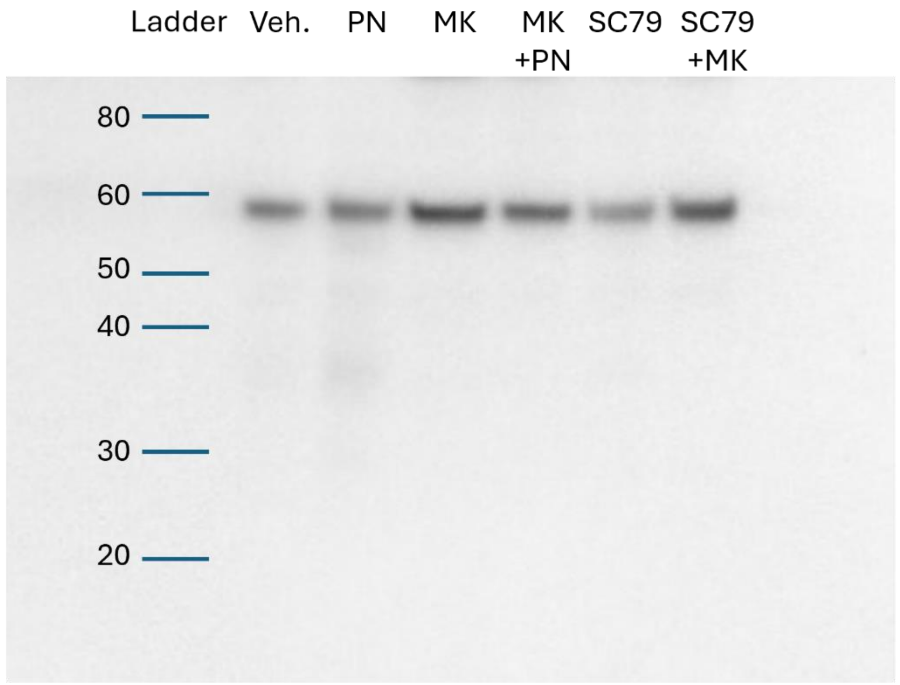

Anti-Akt (Cell Signaling, #9272, 1:1000)

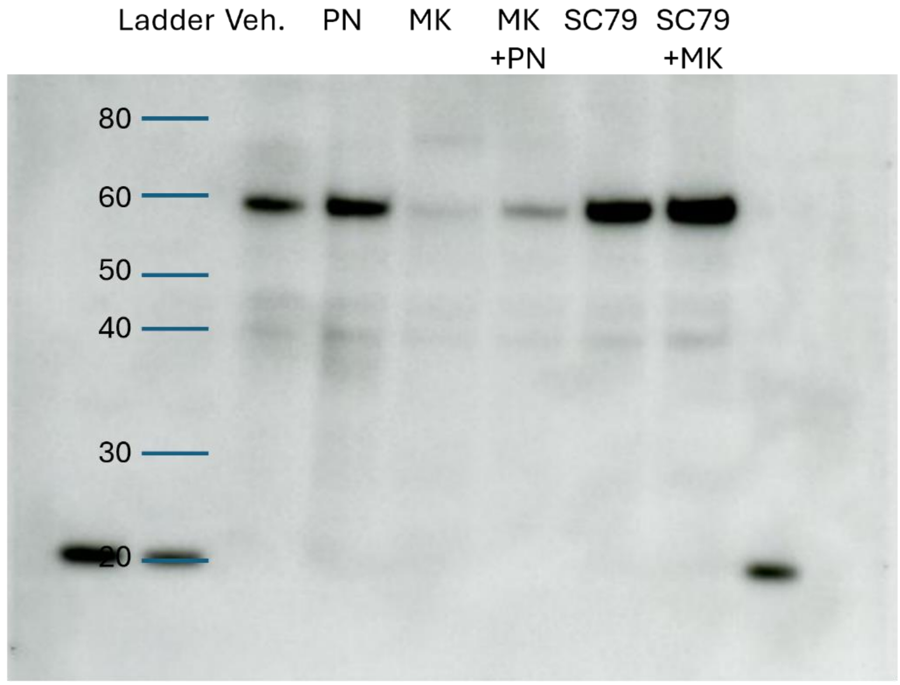

Anti-Phospho-Akt (Ser473) (Cell Signaling, #4060, 1/1000)

Representative unedited western blots from Fig.S4a

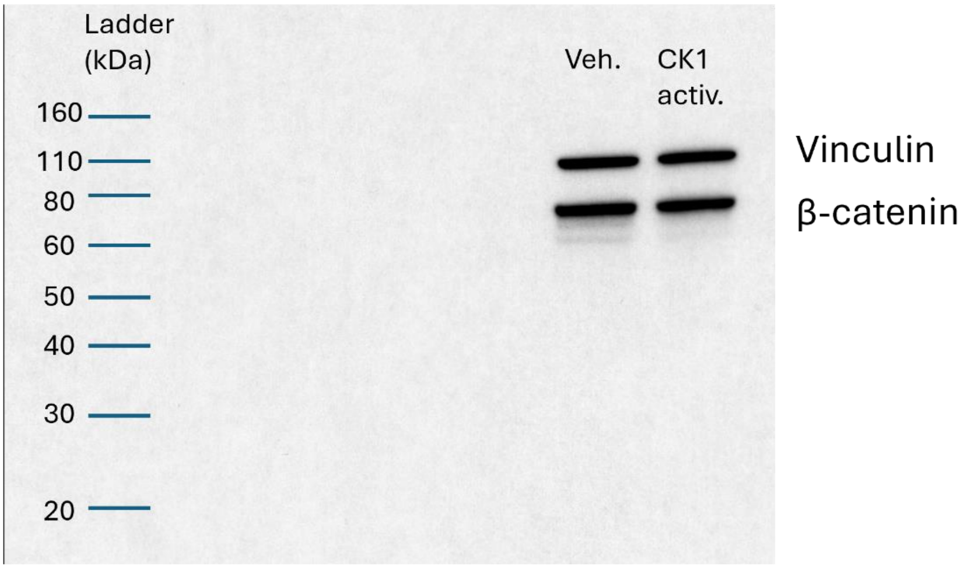

Anti-Vinculin (Cell Signaling, #13901, 1:5000)
Anti-β-catenin (Cell Signaling, #8480, 1/1000)

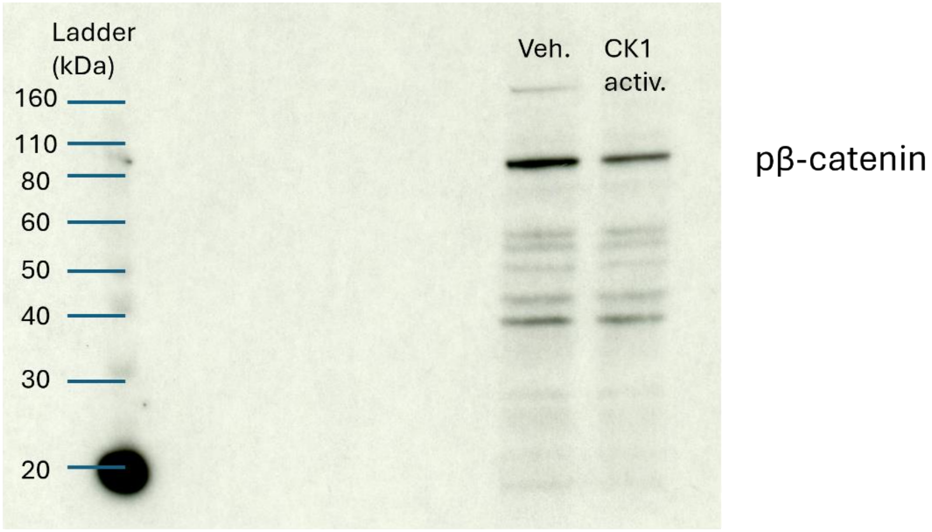

Anti-Phospho-β-Catenin (Cell Signaling, Ser675) (#4176, 1:1000)

All other protein quantification (Fig.4E/Fig.4O) were generated with quantitative capillary immunoassays
